# Exosomes enriched with miR-124-3p show therapeutic potential in a new microfluidic triculture model that recapitulates neuron–glia crosstalk in Alzheimer’s disease

**DOI:** 10.3389/fphar.2025.1474012

**Published:** 2025-03-12

**Authors:** Artemizia Évora, Gonçalo Garcia, Ana Rubi, Eleonora De Vitis, Ana Teresa Matos, Ana Rita Vaz, Francesca Gervaso, Giuseppe Gigli, Alessandro Polini, Dora Brites

**Affiliations:** ^1^ Neuroinflammation, Signaling and Neuroregeneration, Research Institute for Medicines (iMed.ULisboa), Faculty of Pharmacy, Universidade de Lisboa, Lisbon, Portugal; ^2^ Department of Pharmaceutical Sciences and Medicines, Faculty of Pharmacy, Universidade de Lisboa, Lisbon, Portugal; ^3^ Institute of Nanotechnology, National Research Council (CNR Nanotec), Lecce, Italy; ^4^ Dipartimento di Medicina Sperimentale, Università Del Salento, Lecce, Italy

**Keywords:** Alzheimer’s disease modelling, human neural tricultures, microfluidic system, miRNA-engineered exosomes, miR-124-3p mimic, neuron-glia communication, SH-SY5Y cells transfected with APP695

## Abstract

**Background:**

Alzheimer’s disease (AD), a complex neurodegenerative disease associated with ageing, is the leading cause of dementia. Few people with early AD are eligible for the novel Food and Drug Administration (FDA)-approved drug treatments. Accordingly, new tools and early diagnosis markers are required to predict subtypes, individual stages, and the most suitable personalized treatment. We previously demonstrated that the regulation of microRNA (miR)-124 is crucial for proper neuronal function and microglia reshaping in human AD cell models.

**Objective:**

The aim of this study was to develop an efficient miR-124-3p-loaded exosome strategy and validate its therapeutic potential in using a multi-compartment microfluidic device of neuron–glia that recapitulates age-AD pathological features.

**Methods and results:**

Using cortical microglia from mouse pups, separated from glial mixed cultures and maintained for 2 days *in vitro* (stressed microglia), we tested the effects of SH-SY5Y-derived exosomes loaded with miR-124-3p mimic either by their direct transfection with Exo-Fect™ (ET124) or by their isolation from the secretome of miR-124 transfected cells (CT124). ET124 revealed better delivery effciency and higher potent effects in improving the stressed microglia status than CT124. Tricultures of human SH-SY5Y neuroblastoma cells (SH-*WT*) were established in the presence of the human microglia cell line (HMC3) and immortalized human astrocytes (IM-HA) in tricompartmentalized microfluidic devices. Replacement of SH-*WT* cells with those transfected with APP695 (SH-*SWE*) in the tricultures and addition of low doses of hydrogen peroxide were used to simulate late-onset AD. The system mimicked AD-associated neurodegeneration and neuroinflammation processes. Notably, ET124 exhibited neuroprotective properties across the three cell types in the AD model by preventing neuronal apoptosis and neurite deficits, redirecting microglial profiles towards a steady state, and attenuating the inflammatory and miRNA fingerprints associated with astrocyte reactivity.

**Conclusion:**

To the best of our knowledge, this is the first study supporting the neuro- and immunoprotective properties of miR-124-engineered exosomes in a microfluidic triculture platform, recapitulating age-related susceptibility to AD. Our system offers potential to develop personalized medicines in AD patient subtypes.

## Graphical Abstract

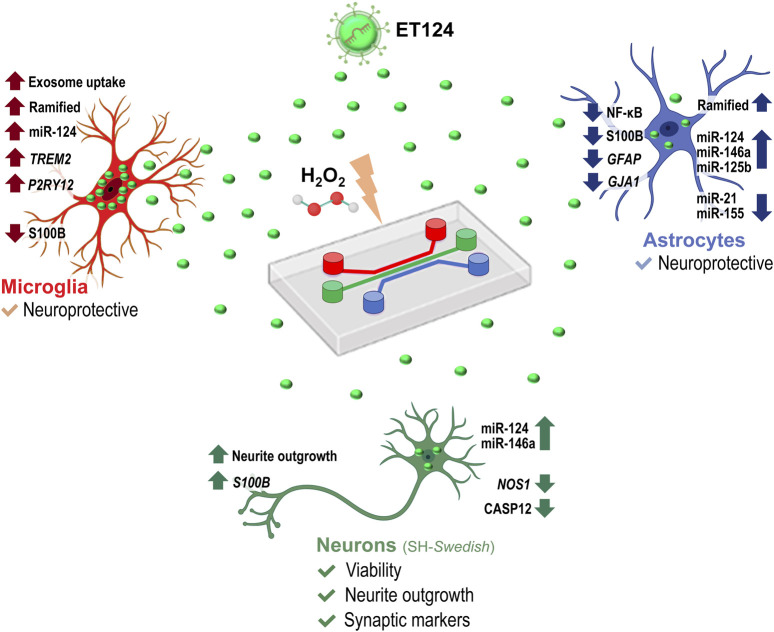

## 1 Introduction

Alzheimer’s disease (AD) is one of the biggest healthcare challenges of the 21st century, standing as the leading cause of dementia. The neuropathological progression typically begins in the temporal lobe and hippocampus, leading to cognitive decline and behavioural disabilities (Kumar et al., 2023). Amyloid plaques (aggregated amyloid beta-peptides*,* Aβ) and neurofibrillary tangles (NFTs) are the most common AD molecular hallmarks, but neuroinflammation, oxidative stress, and pre- and post-synaptic alterations, among other disturbed mechanisms, are equally associated risk factors (Tzioras et al., 2023). Limited therapeutic strategies, successive failed trials, and inaccurate translational AD models indicate that there is an unmet need for disruptive research and that stratification towards selection of patient subtypes is key to identifying and developing innovative and more effective treatments.

Small extracellular vesicles, also known as exosomes, are released from cells and are emerging as promising tools for both diagnostic and therapeutic purposes (Loch-Neckel et al., 2022). These lipid bilayer-bound exosomes, ranging from 30 to 150 nm, play a crucial role in synaptic plasticity and can permeate the blood–brain barrier (BBB), enabling bidirectional communication between the brain and the peripheral system (Yamashita et al., 2018). Exosomes, particularly those isolated from patients with neurodegenerative disorders, may also serve in early diagnosis once they revealed carrying specific biomarkers (Jia et al., 2021). Lately, the potential of exosomes as drug and miRNA mimic/inhibitor delivery systems has been explored for treating neurodegenerative diseases (Cano et al., 2023; Rehman et al., 2023), capitalizing on their low immunogenicity and cytotoxicity, high selectivity, and protection of the encapsulated compound (Kimiz-Gebologlu and Oncel, 2022). Finally, exosomes can be delivered through several administration routes, such as intravenous, subcutaneous, intrathecal, intranasal, intraperitoneal, retro-orbital, intravitreal, and oral routes (Peng et al., 2020; Dong et al., 2021).

Among the several mediators involved in neuro-immune homoeostasis, microRNAs (miRNAs) are a class of non-coding RNAs that can induce post-transcriptional gene silencing (Ponomarev et al., 2013; Brites, 2020). They have been described as key players in dysregulated neuroinflammation associated with the development and progression of neurodegenerative diseases, such as AD (Parisi et al., 2013; Wang et al., 2019). One of the most predominant miRNAs in the CNS (mainly in neurons), with critical nervous and immune functions, is miRNA (miR)-124 (Soreq and Wolf, 2011; Wang et al., 2014). There are conflicting reports regarding miR-124 dysregulation in AD, with some studies on AD patients and models indicating increased levels (Wang et al., 2018; Brites, 2020; Garcia et al., 2021) and others showing decreased levels (Lukiw, 2007; An et al., 2017). We demonstrated that amongst hit inflammatory miRNAs, miR-124-3p is prominent as one of the most involved in the regulation of neuron–microglia paracrine signalling (Brites and Fernandes, 2015; Fernandes et al., 2018; Garcia et al., 2022). Using SH-SY5Y human neuroblastoma cells transfected with the amyloid precursor protein (APP)695 with the *Swedish* mutation (SH-*SWE*) and induced pluripotent stem cell (iPSC)-derived neurons obtained from presenilin1 (*PSEN1*) familial AD patients, similar to *in vitro* models, we showed that miR-124-3p mimic had preventive effects (e.g., inhibition of APP overexpression, of toxic amyloid accumulation, and of phosphorylated tau), while its inhibitor promoted harmful effects (e.g., reduction of dendritic spine density, enhanced APP processing, miR-146a upregulation, and tau phosphorylation) (Garcia et al., 2021). Data corroborate the association of miR-124 with neuroprotective mechanisms (Fang et al., 2012; An et al., 2017; Zhang F. et al., 2022). We also evidenced that neuronal-derived exosomes having miR-124 directly regulate microglia homoeostasis (Fernandes et al., 2018; Garcia et al., 2022), as previously stated for the effects of peripheral administration of miR-124 on the autoimmune encephalomyelitis activated microglia (Ponomarev et al., 2011). miR-124-loaded exosomes were tested in mouse models of several neuropathological disorders (Yang et al., 2017; Esteves et al., 2022; Mavroudis et al., 2023) but still not in AD.

Triculture systems using neurons, astrocytes, and microglia in microfluidic platforms, or in mixed cultures, have been used as models to assess neuron–glia signalling mechanisms as a basis for drug discovery in AD (Park et al., 2018; Guttikonda et al., 2021). However, the therapeutic application of exosomes loaded with miR-124 was never tested in such models.

In the present study, we developed an efficient miR-124-3p-engineered exosome delivery strategy and an improved human neuron–microglia–astrocyte model, allowing autocrine and paracrine signalling. For this, we used a microfabricated multi-compartment device with a fluid-independent circuit (De Vitis et al., 2021). Then, we established an *in vitro* age-AD model by using SH-*SWE* neuroblastoma cells in the presence of human microglia and astrocyte cell lines, under the stress of a low dose of hydrogen peroxide (H_2_O_2_). Finally, we tested exosomes loaded with miR-124 mimic for their ability in preserving AD-associated oxidative damage and ageing, as well as inflammation and neurodegeneration, by sustaining neuron–glial homoeostasis.

## 2 Materials and methods

### 2.1 Animals and ethics statement

For primary cortical microglia, we used mice with the strain B6SJLF1/J purchased from The Jackson Laboratory (Bar Harbor, ME, United States). Maintenance and handling were carried out at the Instituto de Medicina Molecular João Lobo Antunes (IMM) animal house facilities of the Faculty of Medicine, University of Lisbon, Portugal, where the colony was used for breeding. Animals were housed at 4–5 animals/cage, under 12 h light/12 h dark cycle, with room temperature (RT) at 22°C–24°C and 55% humidity, and received food and water *ad libitum*. Experiments were conducted in microglia isolated from the brain cortex of both male and female mouse pups.

All the procedures were carried out in compliance with Portuguese Legislation on Animal Care (Decreto-Lei 129/92, Portaria 1005/92, Portaria 466/95, Decreto-Lei 197/96, Portaria 1131/97) and in agreement with the European Community guidelines (Directives 86/609/EU and 2010/63/EU, Recommendation 2007/526/CE). The protocol was approved by the Institutional animal welfare body, ORBEA-iMM-FFUL, and the National competent autority, the DGAV (Direção Geral de Alimentação Veterinária). Every attempt was made to reduce the number of animals utilized and their suffering in accordance with the 3R principle.

### 2.2 Cortical microglia primary culture

Mixed glial cultures were obtained from the brain cortex of mouse pups, as previously described (Caldeira et al., 2014). In brief, animals were sacrificed at 7–8 days old; their brains were removed from the skull and placed in a Petri dish. The meninges, blood vessels, white matter, and cerebellum were removed under sterile conditions. After tissue homogenization, cells were sequentially passed through 230-μm and 104-μm filters, centrifuged, plated (4 × 10^5^ cells/cm^2^) on uncoated 12-well plates, and maintained with culture medium DMEM-Ham’s F-12 medium (DMEM-F12) supplemented with 2 mM L-glutamine (1%), 1 mM sodium pyruvate (1%), 1× non-essential amino acids (NEAA) (1%), foetal bovine serum (FBS, 10%), and the Antibiotic-Antimycotic (AB/AM) solution (2%) at 37°C, 5% CO_2_ in a Heracell 150 incubator (Thermo Fisher Scientific, Waltham, Massachusetts, United States). Every 7 days, the medium was changed. After 21 days *in vitro* (DIV), microglia were isolated from the astrocyte–microglia mixed culture by gently detaching the top layer of astrocytes with mild trypsinization. Microglia were then maintained until 2DIV, which correspond to a reactive/stressed microglia state, as we previously described (Caldeira et al., 2014). Next, we incubated the microglia with the exosome formulations for 24 h and assessed the delivery efficiency of exosomal miR-124 into the cells, as well as microglia phenotypic markers by RT-qPCR (*Nos2, Arg1,* and Trem2) and immunocytochemistry (Iba1 and P2RY12) assays.

### 2.3 Culture of human cell lines

*Wild-type* human SH-SY5Y (SH-*WT*) cells and SH-SY5Y expressing the APP *Swedish* variant (SH-*SWE*) cells used in this study were a gift from Professor Anthony Turner (Belyaev et al., 2010). Cells were cultured in DMEM, supplemented with 10% FBS and 2% AB/AM in T75 flasks under a humidified atmosphere with 5% CO_2_, at 37°C in a HERA cell 150 incubator (Thermo Fisher Scientific, Waltham, Massachusetts, United States), as performed in our laboratory (Garcia et al., 2021). For differentiation, cells were treated with 10 μM retinoic acid (RA) daily until 7DIV. The human microglial clone 3 cell line (HMC3) was kindly provided by Professor Marc Tardieu (Janabi et al., 1995). HMC3 microglial cells were cultured in DMEM, supplemented with 10% FBS, 2% AB/AM, and 1% L-glutamine in T75 flasks, under a humidified atmosphere with 5% CO_2_ at 37°C, according to the established methods in our laboratory (Fernandes et al., 2018). Immortalized human astrocytes (IM-HA) were purchased from Innoprot (Ref: P10251-IM, Derio, Spain). Cells were cultured in DMEM, supplemented with 2% FBS, 2% AB/AM, and 1% N2 in T75 flasks under a humidified atmosphere with 5% CO_2_ at 37°C. Media from every cell type were changed every other day until reaching 90% confluency. Then, cells were detached by trypsinization under a humidified atmosphere with 5% CO_2_ and pelleted by centrifugation using a Hettich ROTOFIX 32 A centrifuge (Hettich, Tuttlingen, Germany). Pelleted cells were resuspended in the respective media described above and sub-cultured in new T75 culture flasks.

### 2.4 Tricultures in an organ-on-chip microfluidic system

Tricultures were established in tricompartment organ-on-chip (OoC) microfluidic devices, designed and described previously (De Vitis et al., 2021), with minor changes. In brief, the microfluidic device features three perfusable compartments, each measuring 500 μm in width, 250 μm in height, and 6 mm in length, with separate inlets and outlets (diameter of 6 mm). The compartments are connected by a series of tiny, parallel microgrooves, measuring 5 μm in width and 2.5 μm in height, which facilitate the separation of soma and neurites and simultaneously target neurite elongation while allowing direct and paracrine communication between compartments.

The microfluidic device was manufactured using conventional lithography techniques, specifically through a two-step photolithography process and subsequent soft lithography. Initially, silicon substrates measuring 2.5 × 2.5 cm were cleaned with acetone and isopropanol (Sigma-Aldrich, Milan, Italy) and utilized in the first lithography step to create the microgrooves. A uniform layer of photoresist (SU-8 2002, Kayaku Advanced Materials, Westborough, MA, United States) was spin-coated to obtain a resist thickness of 2.5 µm, soft-baked, and then exposed to UV radiation (λ = 365 nm) using a standard mask aligner (MA6, SUSS MicroTec, Garching, Germany). After post-baking, the photoresist was developed in a SU-8 developer solution (Kayaku Advanced Materials) and hard-baked. In the second lithography step, SU-8 2075 (Kayaku Advanced Materials) was spin-coated onto the substrate to achieve a resist thickness of 250 µm. After drying and soft-baking, the substrate was exposed to UV radiation, with a precise alignment of markers included in both the first-level pattern (on the substrate) and the second pattern (on the photomask) using the mask aligner. The substrate was then post-baked, developed, and finally hard-baked. Polydimethylsiloxane (PDMS) replicas were subsequently created by soft lithography using a prepolymer-to-curing agent ratio (Sylgard 184, Dow, Midland, MI, United States) of 10:1. Once polymerized, the cured PDMS layer was punctured with a 6-mm biopsy punch at the inlets and outlets. To assemble the final devices, the microstructured PDMS layer and a clean glass coverslip were treated in oxygen plasma apparatus (PICO low-pressure plasma system, Diener electronic, Ebhausen, Germany) at 100 W, 200 sccm, for 6 s, and heat-treated at 75°C for 4 h to irreversibly bond the two pieces.

SH-*WT* and/or SH-*SWE* neuroblastoma cells, HMC3 microglia, and IM-HA cells were individually seeded into their respective compartments of the microfluidic system, each pre-coated with poly-D-lysine (100 μg/mL) and laminin (4 μg/mL) to promote cell adhesion, after testing other coating formulations (Supplementary Figure S1). The SH-*WT*/SH-*SWE* neuroblastoma cells were plated in the central compartment at a final density of 1 × 10⁶ cells/mL, making up 50% of the total cell population in the triculture setup. These cells were cultured in neural triculture media (NTM), composed of DMEM supplemented with 1% FBS, 2% AB/AM, and 1% N2 supplement. To induce neuronal differentiation, the NTM was further supplemented with 10 μM retinoic acid (RA), and the medium was refreshed daily until 3DIV. On the third day, HMC3 microglia and IM-HA cells were introduced into their respective lateral compartments of the microfluidic device. HMC3 cells were seeded at a final concentration of 4 × 10⁵ cells/mL, constituting 20% of the triculture, while IM-HA astrocytes were seeded at 6 × 10⁵ cells/mL, making up the remaining 30%. This cell ratio of 50% neurons, 30% astrocytes, and 20% microglia showed to be the best tested option (Supplementary Figure S2). After seeding microglia and astrocytes, RA was removed from the NTM media to ensure that all cell types were incubated under the same conditions for an additional 24 h, facilitating cell-to-cell interactions within the triculture system.

At 4DIV, triculture systems were simultaneously exposed to 10 µM H_2_O_2_, to induce oxidative stress and simulate neuronal cell ageing (Chadwick et al., 2010; Ismail et al., 2016). Such a minimal H_2_O_2_ concentration was previously described to not significantly affect the cell viability (Ustyantseva et al., 2022). Concurrently, exosomes transfected with miR-124-3p mimic (referred as ET124) were equally distributed in each cell compartment from the microfluidic system. At 5DIV, after 24 h of treatment, all cells were either fixed using 4% paraformaldehyde (PFA) for subsequent immunofluorescence analysis or separately harvested from each compartment for further molecular analysis via RT-qPCR, providing an enriched sample of each cell type.

### 2.5 Exosome isolation, NTA, and labelling

For neuroblastoma-derived exosome collection, SH-*WT* cells were cultured in media with 1% of FBS (previously depleted in exosomes to prevent contamination). Exosomes were collected from the secretome of cultured SH-*WT* cells at confluency, via differential ultracentrifugation, as optimized in our laboratory (Pinto et al., 2017; Garcia et al., 2022). In brief, equal volumes of cell media were promptly centrifuged at 1000 g for 10 min to pellet cell debris. The supernatants were then centrifuged for 1 h at 16,000 g and filtered through a 0.22-μm pore size membrane, followed by centrifugation at 100,000 g for 2 h in an Ultra L-XP100 centrifuge (Beckman Coulter, Brea, CA, United States). The pellet was resuspended/washed in phosphate-buffered saline (PBS) and (re)centrifuged at 100,000 g for 2 h. The separated exosomes were quantified by nanoparticle tracking analysis (NTA) using the NanoSight instrument (model NS300, Malvern Instruments, Malvern, United Kingdom). Samples were injected into the system under controlled flow using a NanoSight syringe pump and an integrated scripting control system. At least three different videos up to 60-s long were produced, and particle movement was analysed by NTA-software (version 3.1). To track exosome cell internalization upon treatment, exosomes were stained with the PKH67 lipophilic dye. For this, exosomes were resuspended in Dulbecco's phosphate-buffered saline (DPBS) (1:1000) and mixed with an equal volume of PKH67 probe solution for 5 min at RT, using the PKH67 Green Fluorescent Membrane Labelling Kit (Sigma-Aldrich, St. Louis, MO, United States). Labelled exosomes were washed to remove the unbound dye before their addition to the cell cultures (Pinto et al., 2017).

### 2.6 Exosome transfection with Exo-Fect™ for miR-124-exosome loading

Before transfection, each exosome batch was individually characterized in terms of particle concentration and total exosomal protein content using NTA and Bradford assay, respectively. This characterization allowed for a precise quantification, estimating approximately 2.20 ± 1.54 × 10⁷ exosomes per μg of exosome protein to be used in each experimental setup. This was particularly relevant since the same number of exosomes not always corresponded to the same protein content. Therefore, we normalized for both the number and protein content.

To load neuroblastoma cell-derived exosomes with the miR-124-3p mimic (Ambion, Austin, TX, United States), we utilized the Exo-Fect™ Exosome Transfection Kit (Systems Bioscience, Palo Alto, CA, United States), according to manufacturer instructions, with minor changes. Transfection started by mixing 2 nmol of miR-124-3p mimic with 10 μL of the Exo-Fect reagent in a transfection tube, creating a total reaction volume of 30 μL. This mixture was gently vortexed and incubated at room temperature for 10 min to allow for optimal formation of the miRNA–reagent complex. Subsequently, 120 μL of freshly isolated exosomes from neuroblastoma cells, previously quantified and characterized, were added to the reaction mixture, increasing the total volume to 150 μL. The mixture was then incubated at 37°C for 10 min to facilitate the incorporation of miR-124-3p into the exosomes. During this incubation, the tube was manually agitated every 2 min to enhance the efficiency of the transfection process. To stop the transfection, 30 µL of the ExoQuick-TC reagent was added to the tube, which was then placed in ice for 30 min. To isolate exosomes, the tube sample was centrifuged at 14,000 rpm for 3 min. The supernatant was carefully removed, and the pellet containing the miR-124-3p-loaded exosomes (referred to as ET124) was then resuspended in 100 µL of sterile PBS. Successful loading of miR-124-3p into the exosomes was verified by RT-qPCR, utilizing specific predesigned primers for miR-124-3p (Supplementary Table S1). ET124 were immediately labelled with the PKH67 dye, as abovementioned (see the Exosome isolation, NTA and labelling section), and used as soon as possible to ensure highest fluorescence intensity for exosome tracking and their cell internalization in the different assays.

### 2.7 Evaluation of the neural cell viability by the nexin assay

To determine the viability of SH-*WT*/SH-*SWE* neuroblastoma cells, microglia, and IM-HA astrocytes in the triculture microfluidic system, in the absence or presence of H_2_O_2_, and before or after exosome treatment, the cells were detached by trypsinization and spun down at 500 g for 5 min. Pellets were resuspended in 1% bovine serum albumin (BSA) in PBS and stained with phycoerythrin-conjugated annexin V (V-PE) and 7-amino-actinomycin D (7-AAD), using the Guava Nexin Reagent^®^ (Merck Millipore, Burlington, MA, United States). Stained cells were analysed using a flow cytometer (Guava easyCyte 5 HT, Merck-Millipore), operated by Guava Nexin software. Three cellular populations were distinguished in the nexin assay: viable cells (annexin V-PE and 7-AAD double-negative), early apoptotic cells (annexin V-PE positive and 7-AAD negative), and late apoptotic/necrotic cells (annexin V-PE and 7-AAD double-positive).

### 2.8 RT-qPCR assay

Primary cortical microglia were washed once with PBS and collected in TRIzol™ (Thermo Fisher Scientific, Waltham, Massachusetts, United States) for RNA extraction.

In the microfluidic triculture system, samples were collected by consecutively streaming TRIzol™ into each cell compartment, in a separate way for each cell type. This allowed us to collect neuronal, microglial, and astrocyte samples from the same microfluidic system, with a minimal cross-contamination of the cellular content.

Total RNA was extracted, according to the manufacturer’s instructions, and quantified, as performed in our laboratory (Garcia et al., 2022). The NanoDrop ND100 Spectrophotometer (NanoDrop Technologies, Wilmington, United States) was used for RNA quantification.

For miRNA assessment, 5 ng/μL of total RNA was converted into cDNA using the miRCURY LNA™ RT Kit (QIAGEN) under controlled conditions: 60 min at 42°C, followed by heat inactivation of reverse transcriptase for 5 min at 95°C and subsequent cooling to 4°C for 24 h. RT-qPCR was performed on a QuantStudio 7 Flex Real-Time PCR System (Applied Biosystems, Life Technologies), using Power SYBR™ Green PCR Master Mix with the pre-designed primers listed in the Supplementary Table S1. RT-qPCR was done in 384-well plates with each sample measured in duplicate and non-template controls (NTCs) included for each amplification product. U6 and SNORD110, two reference genes, were used to normalize miRNA expression. The RT-qPCR conditions were as follows: polymerase activation/denaturation at 95°C for 10 min, followed by 50 amplification cycles at 95°C for 10 s and 60°C for 1 min (a ramp rate of 1.6°/s). Specificity of the amplified products was confirmed by melting curve analysis.

Regarding gene expression, 1000 ng/μL of total RNA was converted into cDNA using the Xpert cDNA Synthesis Supermix Kit (GRiSP, Porto, Portugal), according to the manufacturer’s instructions at optimized conditions: 37°C for 15 min, 60°C for 10 min, and 95°C for 3 min. Subsequently, template cDNA was amplified by quantitative RT-qPCR using the Xpert Fast SYBR Mastermix BLUE Kit (GRiSP, Porto, Portugal), using the primer sequences indicated in Supplementary Table S2. RT-qPCR was performed in 384-well plates with each sample measured in duplicate and NTCs included for each amplification product. β-actin was used as an endogenous control to normalize gene expression levels. Running conditions for the RT-qPCR were as follows: 50°C for 2 min followed by 95°C for 2 min and 40 cycles at 95°C for 5 s and 62°C for 30 s. After amplification, the specificity of the amplified products was verified by melting curve analysis. Both miRNA and mRNA RT-qPCRs were run on the QuantStudio 7 Flex Real-Time PCR System (Thermo Fisher Scientific, Waltham, Massachusetts, United States).

Relative mRNA/miRNA expression levels were measured using the ΔΔCT method relative to the respective endogenous control, as previously published (Garcia et al., 2022). Normalized results were expressed as Log_2_ (2^−ΔΔCT^) vs. the respective control sample.

### 2.9 Exosome protein quantification using the Micro BCA™ Protein Assay Kit

Total protein was extracted from concentrated exosome samples using a modified version of RIPA buffer [2% sodium deoxycholate; 20 mM HEPES buffer; 200 mM KCl; 1 mM EDTA pH 8.0; 0.2% sodium dodecyl sulphate (SDS); 20% glycerol and H_2_O MilliQ]. Samples were diluted at least 20 times to prevent the buffer from interfering with the Micro-BCA™ Protein Assay Kit (Thermo Fisher Scientific, Waltham, Massachusetts, United States), following the manufacturer’s recommendations. Protein concentration was measured using a Varioskan LUX multimode plate reader (Thermo Fisher Scientific™, Waltham, MA, United States). Absorbance was read at 562 nm.

### 2.10 Immunocytochemistry assay

For immunocytochemistry, primary microglia and neuroblastoma cells (referred to as neurons hereafter) were cultured in 12-well plates containing HCl-washed coverslips, while the microfluidic devices with neurons + microglia + astrocytes were directly used as the assay platform. In brief, primary cortical microglial cells and tricultures were fixed with 4% (w/v) paraformaldehyde in PBS for 20 min. Then, cells were permeabilized with 0.2% Triton X-100 in PBS for 10 min and further blocked with 3% BSA in PBS for 30 min. Afterwards, the cells were incubated with primary antibodies (Supplementary Table S3) at 4°C and overnight, as usual in our laboratory (Vaz et al., 2021). All antibodies were diluted in PBS (1% BSA). In the following day, cells were incubated with species-specific secondary antibodies (Supplementary Table S3) for 2 h. Coverslips were mounted into a glass in Fluoromount-G to be visualized by confocal microscopy. An extra washing step was performed by the addition of DPBS in the microfluidic device, followed by the application of a 40 µL drop of Fluoromount-G (Merck-Millipore) in each cell compartment.

### 2.11 Confocal microscopy analysis

Confocal fluorescence z-stack images of primary microglia and microfluidic cell triculture chips were acquired (under the navigator mode) using a Leica TCS SP8 inverted microscope (Leica Microsystems, Wetzlar, Germany), both with ×10 and ×40 (oil immersion) objectives, sequential laser excitation at 405/488/552/638 nm, and spectral detection adjusted for the emissions of AlexaFluor 405/488/594/647 dyes, respectively. The equipment was controlled by Leica LAS X software (Leica Microsystems).

### 2.12 Post-acquisition image treatment and analysis

Image treatment, including concatenation, and z-stack analysis (maximum fluorescence intensity) were performed using Fiji software (Schindelin et al., 2012). Pixel-integrated single-cell multiparametric analysis was done in AIVIA software v.12.0.0 (Leica Microsystems), using the following 2D recipes: neurite outgrowth; cell analysis, and particle count. Settings were adjusted for the identification of single cells, accordingly with each measurement requirements. In the images of the microfluidic cell triculture system, different regions of interest (ROIs) were created to perform a personalized analysis adjusted to the cell type of each compartment.

### 2.13 Statistical analysis

Statistical analyses were performed with GraphPad Prism 9 (GraphPad Software Inc., San Diego, CA, United States). All statistical comparisons were performed using one-way ANOVA when each dataset met the required assumptions for ANOVA, including homogeneity of variances and normality. Tukey’s *post hoc* test was applied to determine statistical significance between experimental groups. Only *p* < 0.05 was considered statistically significant. Results from a minimum of three independent experiments (*n* = 3) are expressed as mean ± SEM. Individual replicates were visually represented in each graph, allowing for easy verification of the sample sizes and distribution in each condition. In the triculture experiments, statistical analyses were carried out separately for neurons, microglia, and astrocytes. Whenever found, outliers were removed from the datasets to ensure the accuracy and robustness of the statistical analyses. For correlation analyses, RT-qPCR data were analysed using SRplot (Tang et al., 2023), including the basic correlation plot package (https://www.bioinformatics.com.cn/plot_basic_corrplot_corrlation_plot_082_en). Bivariate Pearson’s correlation coefficients were calculated, and their significance was considered whenever *p* < 0.05. Raw data correlation matrix, Pearson correlation coefficients (R^2^), and *p*-values are provided in Supplementary Datasheet S1.

## 3 Results

### 3.1 Exo-Fect™ exosomal transfection with miR-124-3p mimic reveals high efficiency and steers a neuroprotective phenotype on stressed microglia

Emerging studies have suggested that dysregulation of miR-124 is related to the pathogenesis of neurodegenerative diseases, such as AD (Han et al., 2019). Therefore, regulation of miR-124 expression may represent a promising therapeutic approach in patients where its downregulation is associated with the onset and progression of the disease. Indeed, we have previously showed that miR-124-3p regulation with its mimic counteracted AD neuronal impairments and restored microglial homoeostasis (Garcia et al., 2021; Garcia et al., 2022). Considering that exosomes are the main natural carriers of miRNAs (Loch-Neckel et al., 2022), we anticipated that delivery of exosomes loaded with miR-124 mimic could represent a promising strategy to recover homoeostatic neuron–glia balance in AD patients with defective neuronal values of miR-124.

**FIGURE 1:**
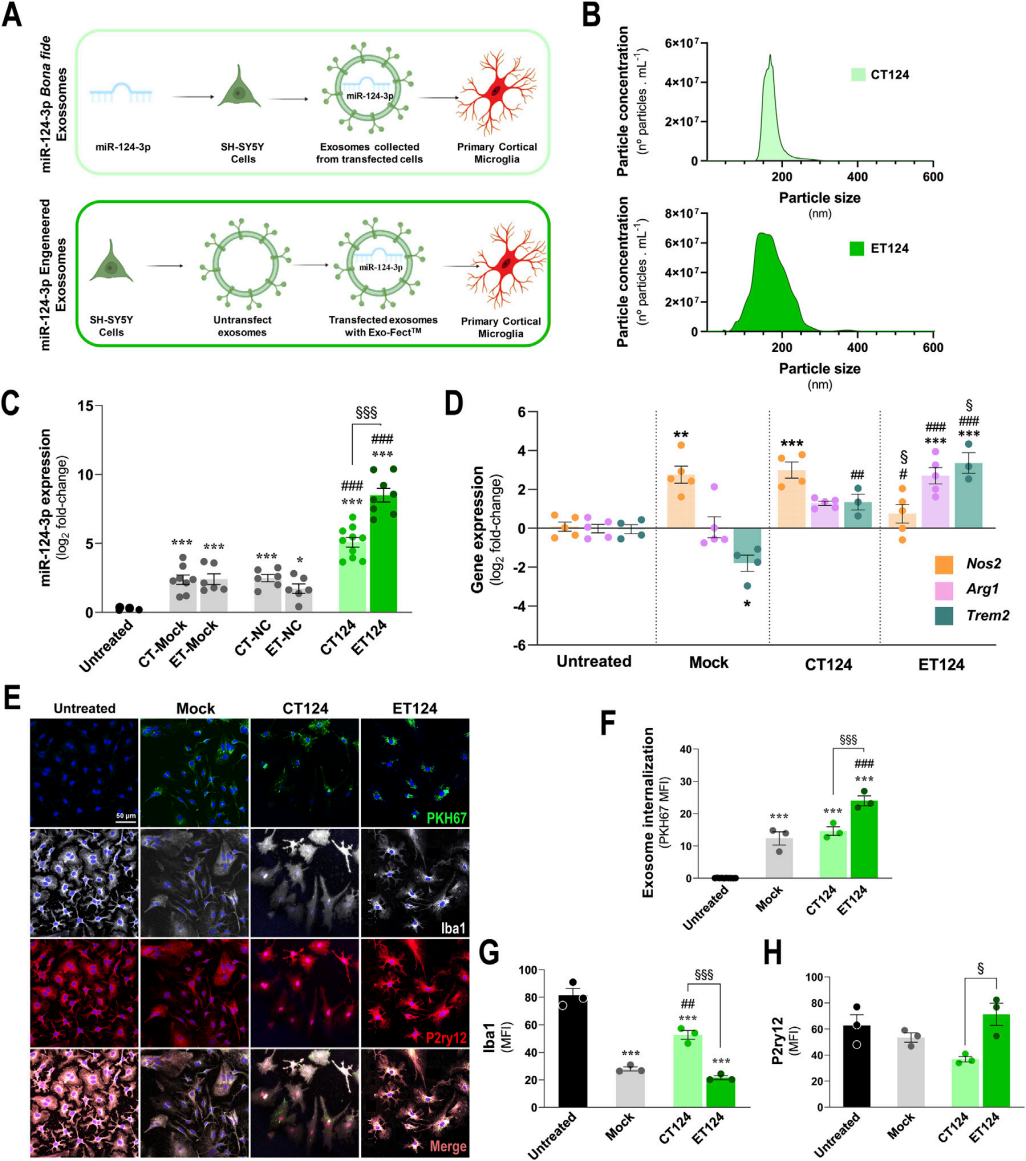
Comparative transfection processes, exosome delivery, and microglial targeting efficiencies of miR-124-engineered exosomes upon cell- and exosomal-enriched miR-124 strategies. CT124, obtained by transfecting SH-*WT* cells with the miR-124 mimic, followed by secretome collection and exosome isolation. ET124, obtained by separating exosomes from the secretome of SH-*WT* cells, followed by direct Exo-Fect transfection with the miR-124 mimic. **(A)** Schematic representation of the tested strategies for miR-124-3p exosome enrichment and delivery on cortical 2DIV stressed microglia. **(B)** Representative histograms of the size and concentration of CT124 and ET124 exosomes. **(C)** miR-124-3p expression levels in microglia treated with ET124 and CT124. **(D)** Expression levels of microglial *Nos2*, *Arg1*, and *Trem2* in each tested condition after exosome delivery. Both miRNA and gene expressions were determined by RT-qPCR. **(E)** Fluorescent images of microglia immunostained for Iba1 and P2RYy12, after incubation with PKH67-labelled CT124 and ET124. Internalization of PKH-67 labelled exosomes by microglia **(F)**, Iba1 **(G)** and P2ry12 **(H)** mean immunofluorescence intensities (MFI, arbitrary units), in each tested condition. Data are mean ± SEM, from at least n = 3 independent experiments. ***p < 0.001, **p < 0.01, and *p < 0.05 vs. untreated condition; ^###^ *p* < 0.001, ^##^ *p* < 0.01, and ^#^ *p* < 0.05 vs. mock; ^§§§^ *p* < 0.001, ^§§^ *p* < 0.01, and ^§^ *p* < 0.05 vs. CT124 cells; one-way ANOVA with Tukey's *post hoc* test. Microglia (human CHME3 cell line); Neuroblastoma SH-*WT*, SH-SY5Y *wild-type* cells; *Arg1*, arginase 1; Iba1, ionized calcium binding adaptor molecule 1; *Nos2*, inducible nitric oxide synthase-coding gene; P2ry12, purinergic receptor p2y12; *Trem2*, triggering receptor expressed on myeloid cell 2 coding gene.

Based on such background, we used two different loading strategies of miR-124 in neuronal exosomes, as schematized in Figure 1A, and as follows: one was the transfection of SH-*WT* cells with the miR-124-3p mimic and subsequent isolation of the released miR-124 enriched exosomes (CT124); and the other was the direct transfection of the miR-124-3p mimic in exosomes previously isolated from SH-*WT* cell secretome using the Exo-Fect™ kit (ET124). Then, CT124 and ET124 were first compared relatively to size distribution and particle count using the NTA assay, as described in the methods section (Figure 1B). No differences were found between CT124 and ET124 for the number and size, although ET124 revealed an enlarged variation in the exosome diameter size (from 100 to 200 nm). Next, we assessed CT124 and ET124 delivery efficiencies in the 2DIV cortical *WT* mouse microglia, by determining the microglial content in miR-124 vs. non-exosomal treated cells (untreated), mock-transfected (mock), and negative controls (NCs) (Figure 1C). Both approaches (CT124 and ET124) significantly upregulated microglial miR-124-3p vs. mock-transfected exosomes (p < 0.001) and vs. negative control-transfected exosomes (p < 0.001). Nevertheless, ET124 had a more efficient delivery (20-fold, *p* < 0.001), when compared to CT124.

Importantly, the neuroblastoma-derived exosomes used as mock and negative controls revealed to be natural miR-124-3p carriers, as we have previously demonstrated (Garcia et al., 2022). Based on the similar results of mock and negative controls, in the subsequent experiments, we used only the mock control.

To further evaluate how far CT124 and ET124 were able to regulate the originally stressed 2DIV microglia, we assessed core activity state biomarkers, namely, the genes encoding the nitric oxide synthase (*Nos2*), arginase 1 (*Arg1*), and the triggering receptor expressed on myeloid cells (*Trem2*). We also noted that exosomes, as we previously demonstrated (Pinto et al., 2017), can *per se* also promote microglial activation. This was the case for the increased *Nos2* (*p <* 0.01) and the decreased *Trem2* (*p <* 0.05) in mock samples relatively to non-exosomal treated cells (untreated), as shown in Figure 1D. Addition of CT124 was not able to significantly decrease *Nos2* or revert *Arg1* (despite the slight elevation) levels but significantly enhanced *Trem2* expression values, suggesting a “calming” effect and recovery of microglial metabolic fitness. More prominent effects were obtained for ET124 in mitigating *Nos2* overexpression relatively to both CT124 and mock samples (Figure 1D, *p <* 0.05, for both), as well as on upregulating *Arg1* and *Trem2*, as compared with both untreated and mock controls (*p <* 0.001, for both). Importantly, the ability to enhance the microglial phagocytosis rate was mainly sensed for ET124 and barely for CT124, when considering the *Trem2* overexpression (*p* < 0.05) and the trend towards *Arg1* upregulation (*p* = 0.06).

Given these promising results, we investigated whether such effects were caused by an increased exosome uptake of ET124 vs. CT124 by microglia. Correspondingly, we labelled exosomes (mock control, CT124, and ET124) with the PKH67 lipophilic dye to monitor and compare exosome internalization by the microglial cells. Although all exosomal formulations were successfully internalized by the 2DIV stressed cortical microglia, the uptake of ET124 was markedly higher (*p* < 0.001) than that of CT124 and mock control, as shown in Figures 1E, F.

Iba1 and the purinergic receptor P2Y12 (P2RY12) are considered specific and complementary microglia markers once they are not individually expressed by all microglia. Some subsets express Iba1, and some others lose P2RY12, as observed for the microglia around Aβ plaques (Kenkhuis et al., 2022). This means that they should be evaluated in combination, probably reflecting diverse functional phenotypes. Therefore, we assessed both markers in the microglia treated with CT124 and ET124 (Figures 1G, H) by immunocytochemistry. Our results suggest that the subtype induced by CT124 is considerably different from that triggered by ET124. Iba1 was sustained by CT124, with higher levels than mock (*p <* 0.01) or ET124 (*p <* 0.001), which showed lower levels than the untreated microglia (*p <* 0.001). On the contrary, untreated, mock, and ET124 experiments revealed between 60% and 70% of microglial positive cells for P2ry12 but only 40% in the case of the cells treated with CT124. The loss of Iba1 immunostaining seems to not be due to ET124, once it was also observed for the mock control. However, the loss of P2ry12 was determined only by CT124.

Together, such findings attest the higher efficiency of the exosomal direct transfection of miR-124-3p with Exo-Fect™ and suggest that ET124 redirects microglia into a more neuroprotective phenotype than CT124. Hereafter, the experiments were conducted only with ET124.

To later explore the potential neuroprotective properties of ET124 in a model recapitulating the homoeostatic imbalance associated with AD pathology, we developed a system with dynamic microglia–neuron–astrocyte tricultures in a microfluidic device, allowing paracrine signalling, and investigated the cell type-specific distribution of ET124 using the SH-*SWE* cells and H_2_O_2_ in the presence of human astrocytes and microglial cell lines. In this way, we envisioned to recreate the conditions of neurodegeneration, oxidative stress, neuroinflammation, and ageing usually linked to the onset and progression of AD.

### 3.2 Establishment of a microfluidic multicompartment system for sustained microglia–neuron–astrocyte crosstalk

**FIGURE 2:**
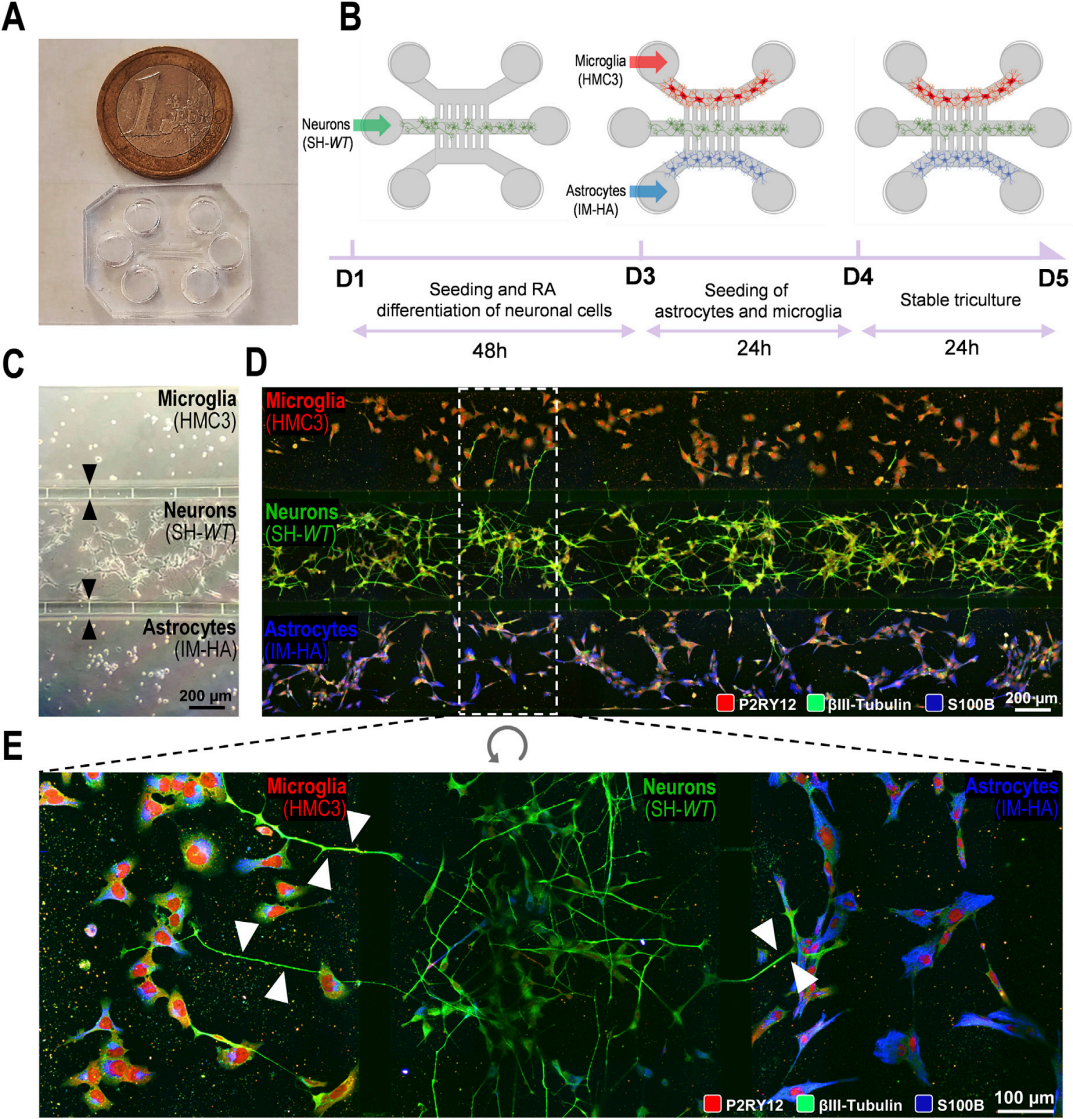
Generation and optimization of a 2D triple co-culture of neural cells (human neurons, microglia, and astrocytes) on a microfluid chip system to investigate paracrine signalling by neurites and cell-derived extracellular vesicles and soluble factors. **(A)** Photography of a microfluidic chip and size comparison to 1€ coin. **(B)** Schematic illustration of the experimental design implemented for microglia–neuron–astrocyte tricultures along the days in culture (day 1, D1 until the ending day 5, D5. **(C)** Phase contrast microscopic image of the three human cellular types (neuroblastoma SH-SY5Y cell line as neurons; CHME3 microglia cell line as microglia; and immortalized astrocyte cell line, IM-HA, as astrocytes) cultured in the microfluidic device, with intercellular communication via the microchannels with 5 µm in width and 50 µm in length (indicated by black arrows). **(D)** Fluorescent images of microglia (on the top compartment), neurons (on the middle compartment), and astrocytes (in the bottom compartment) cultured in the microfluidic device, evidencing immunostaining of microglial P2RY12 (red), of neuronal βIII-tubulin (green) and of astrocytic S100β (blue) expression predominance. **(E)** Inset image from panel D showing neurite transmigration from neuronal to both glial cell compartments through the 5-µm microchannels (indicated by white arrows). P2RY12, purinergic receptor P2Y12; S100B, S100 calcium-binding protein Β.

Although microfluidic devices have been proposed for human triculture systems modelling neurodegeneration (Park et al., 2018; Amartumur et al., 2024; De Vitis et al., 2024), to the best of our knowledge, the present work is original in assessing ET124-based therapeutics to target neurodegeneration and neuroinflammation without cell-to-cell direct interactions. For this, we initially developed a triculture in a multicompartment microfluidic device, where three different human neural cell populations (neurons–astrocytes–microglia) were cultured in a fluidically dependent closed circuit of secretome (De Vitis et al., 2021) (Figure 2A). With this system, it is possible to follow the temporal-dependent paracrine signalling exerted all together by each cell type through their released soluble factors and extracellular vesicles. Considering the contradicting literature with different technical approaches for establishing triculture systems (Park et al., 2018; Guttikonda et al., 2021), we tested multiple procedures to optimize the cell triculture stability by defining the best setup to support each cell type (Supplementary Figure S1 for defining the cell coating; Supplementary Figure S2 for defining the best neuron–astrocyte–microglia cell ratio). We have decided to use a neuron–astrocyte–microglia ratio of 5:3:2, considering the better results achieved. It is noted that the interaction between the three cell types seems to improve the differentiation of SH-SY5Y cells, used as neuron-like cells.

We started by selecting the neural cell type to be plated in the system. For the neurons, we used neuron-like SH-*WT* differentiated cells because we are familiar with these cells, they are not expensive, and they are easy to use. They were plated in the middle compartment of the microfluidic device and differentiated for 2DIV (D1–D3, 48 h) with RA (Figure 2B). At 3DIV (D3), the human HMC3 cell line and the immortalized human astrocytes were plated on the top and bottom compartments, respectively, until 5DIV (D5). Cell images by phase contrast microscopy are shown in Figure 2C, upon the completion of the tricultures at D5. Here, we used devices with shorter microchannels (50 µm) across the cell compartments, to more easily follow the neurite extension between compartments using the fluorescence microscope and the immuno-specificity for βIII tubulin in neuronal cells (Figure 2D). Immunostaining of microglial P2RY12 (red), neuronal βIII-tubulin (green), and astrocytic S100 calcium-binding protein B (S100B, blue) expression predominance can be clearly identified in Figure 2E. Each cell type remained inside their specific compartment, except for neuronal neurites (visible in green) that transmigrated into the glial compartments through the narrow channels (white arrows), as shown in Figure 2E. Culture optimization was also validated in tricultures using SH-*SWE* cells, instead of SH-*WT* cells (data not shown).

Results confirm the success in establishing a viable neural cell triculture without cell-to-cell direct contact, reaching a state of equilibrium where the neurite outgrowth and the released secretome, enriched in cell-derived soluble factors and extracellular vesicles, contribute to a balanced signalling dynamics with paracrine and autocrine responses. The system will be fundamental to recreate a late-onset AD model (aged-related AD), a requisite to evaluate the ET124 cell-specific distribution and their subsequent neuroimmune modulatory effects in each neural cell type.

### 3.3 Microglia show the highest uptake of ET124 in the dynamic triculture microfluidic system recapitulating AD under stressful and ageing conditions

**FIGURE 3:**
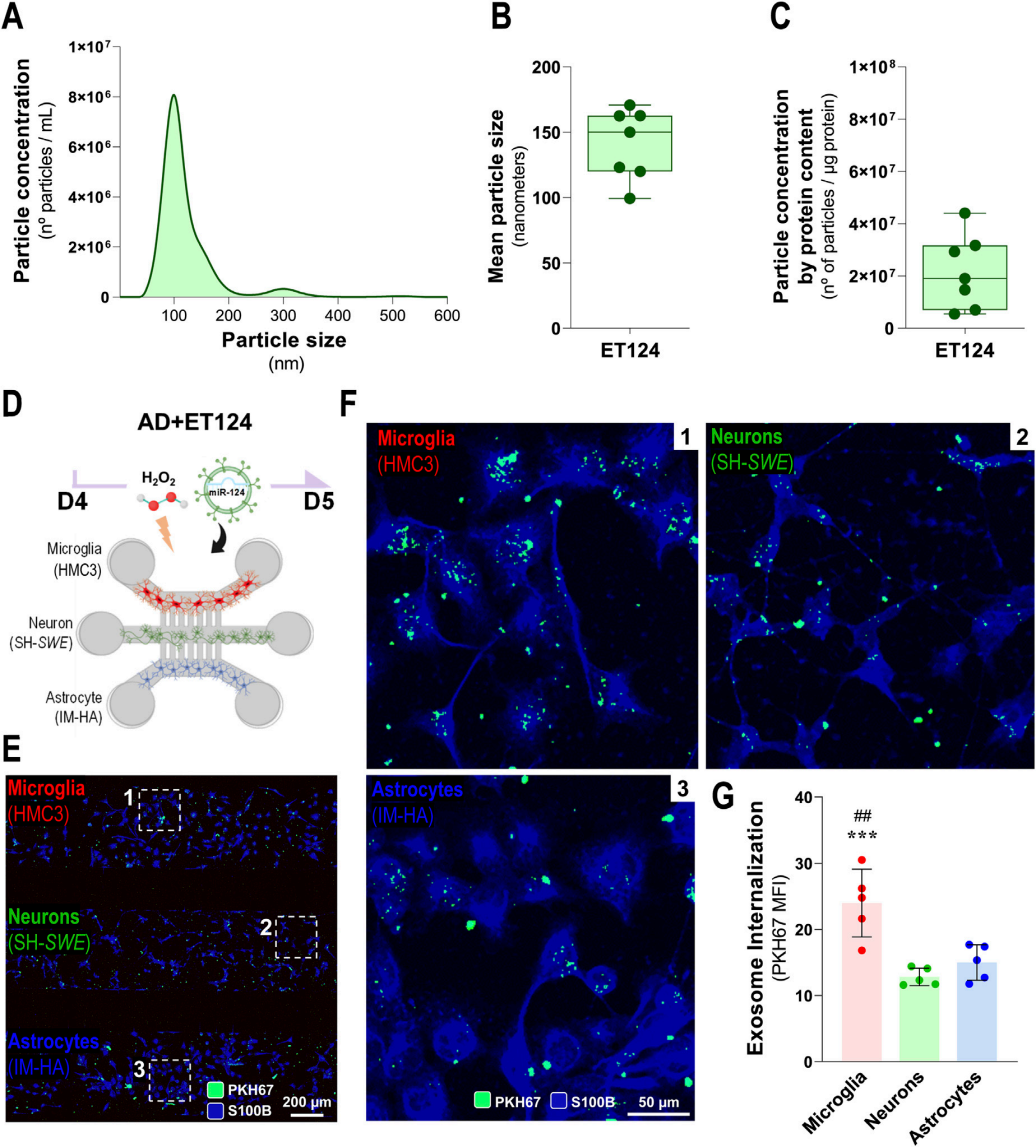
Characterization of ET124 for the size, number, and concentration and assessment of their delivery into microglia, neurons, and astrocytes in the AD-like microfluidic triculture model, after exosome labelling. **(A)** Representative histogram of ET124 for the particle size (nm) and number (particles/mL) by Nanosight tracking analysis (NTA). **(B)** Mean particle size of ET124 used in experiments. **(C)** Average ratio of particle number per microgram (μg) of exosomal protein used in experiments. **(D)** Schematic representation of the AD-like triculture model used in the experiments, with device microchannels of 5 µm diameter and 250 µm long. **(E)** Fluorescent images of the distribution of PKH67-labelled ET124 in the tricultures all positive for S100B with insets for zoomed-in images **(F)**, which reveal the internalization of PKH67-labelled ET124 by microglia (CHME3, **1**), neurons (SH-*Swe*, **2**), and astrocytes (IM-HA, **3**). **(G)** Pixel-integrated single-cell quantification of the mean fluorescent intensities (MFI, arbitrary units) for the number of PKH67-positive vesicles internalized by each cell type (from each cell compartment), as indicated in the Materials and methods section. Data are mean ±SEM, from at least three independent experiments. ***p < 0.001 vs. neurons and ^##^ *p* < 0.01 vs. astrocytes by one-way ANOVA with Tukey's *post hoc* test. AD, Alzheimer’s disease. ET124, exosomes isolated from the SH-*WT* cell secretome after transfection with miR-124-3p mimic; SH-*WT, wild-type* human SH-SY5Y cells; neurons, SH-*SWE*, SH-SY5Y cells expressing the APP *Swedish* variant; Microglia (human CHME3 cell line); astrocytes; IM-HA, immortalized human astrocytes; S100B, S100 calcium-binding protein B; PKH67, green, fluorescent membrane linker.

Tricultures were established as indicated in Section 2.2 but now using the SH-*SWE* cells instead of SH-*WT* cells to induce cell environmental pathology mediated by homoeostatic imbalance in the microglia–neuron–astrocyte cultures. We have previously demonstrated that SH-*SWE* cells release inflammatory mediators, mature and immature amyloid precursor protein (APP), and soluble APP alpha (sAPPα), as well as exosomes with an elevated cargo of inflammatory miRNAs that mimic their donor cells (Fernandes et al., 2018). In addition to the differentiation of the cells with RA, we used a low dose (10 µM) of H_2_O_2_ to trigger a viable neural phenotype in-part reminiscent of aged or damaged neuron derived from oxidative stress and neuroinflammation induction (Chadwick et al., 2010; Gunawardena et al., 2019), thus recreating our aged-related AD-like model before exposure to ET124 (Figure 3). Indeed, such conditions are known to be associated and further aggravate AD pathology (van Rensburg et al., 1997; Milton, 2004; Wang et al., 2023).

As shown in Supplementary Figure S3, the addition of H_2_O_2_ at 10 µM to SH-*SWE* cells in monoculture produced a dramatic reduction of total neurite length (*p <* 0.001), relatively to the SH-*WT* matched cells. Importantly, it caused a reduction of miR-124-3p expression levels, which was more prominent in SH-*SWE* cells when compared to SH-*WT* cells (*p* < 0.01 vs. untreated). These findings recapitulate a subtype of AD patients with defective miR-124 expression—which is our model of choice to test ET124 reparative benefits.

In previous studies from our group, exosomes isolated from SH-*WT* cells were characterized in terms of morphology by transmission electron microscopy (TEM), protein markers by Western blot, and number/size distribution by NTA (Fernandes et al., 2018; Garcia et al., 2021). Such exosomes typically showed a spherical morphology with the usual cup-shaped distortion by surface desiccation by TEM in high magnification, as well as the predictable presence of ALIX, CD63, and Flotillin-1 proteins. Therefore, in the present manuscript, we only characterized ET124 (obtained as previously described) in terms of concentration, size, and particle concentration per µg protein. Most of the samples of ET124 presented an average of 8 × 10^6^ particles per mL (Figure 3A) and showed a mean particle size between 120 and 160 nm (Figure 3B). To obtain reproducibility among the experiments, we used both the number of exosomes and the exosomal protein concentration to estimate the amount of ET124 to be added to each microfluidic triculture experiment. Indeed, the same number of exosomes may not represent the same concentration, which is why we used a ratio between the number of particles and the protein content (number of particles/µg of protein, Figure 3C). An average of 2.16 ± 1.41 × 10^7^ particles per μg of exosomal protein were administered to each triculture, divided in three doses equally distributed by individual cell compartment.

Before separately evaluating some of the resultant effects of the addition of ET124 in our AD-like triculture model, which will be addressed further, we tracked PKH67-labelled ET124 internalization by microglia (HMC3), neurons (SH-*SWE*), and astrocytes (IM-HA) under H_2_O_2_ treatment (Figures 3D, E). Here, we used the microfluidic devices with intercellular communication via the five longer inter-compartment microchannels (250 µm) to prevent the interference of neurites in the exosome cell distribution. Cells were stained for S100B to allow common but uncompartmentalized cell visualization. The pixel-integrated quantification of fluorescent vesicles permitted their counting by AIVIA software. Images show that ET124 were internalized by all cell types (Figure 3F) with a significantly higher engulfment by microglia (*p <* 0.001 vs. neurons; *p <* 0.01 vs. astrocytes, Figure 3G). This is not without precedent if we consider microglia predominant phagocytic function and our previous data favouring microglial exosome uptake (Pinto et al., 2017; Garcia et al., 2022). As far as this ability to uptake ET124 will be reflected in more extended reparative effects on microglia than on astrocytes and neurons in the AD-like model, it will be a matter of evaluation at a later stage.

### 3.4 Delivery of ET124 into the microfluidic-based AD triculture model counteracts neuronal apoptosis and caspase-12 activation, as well as glial activation

**FIGURE 4:**
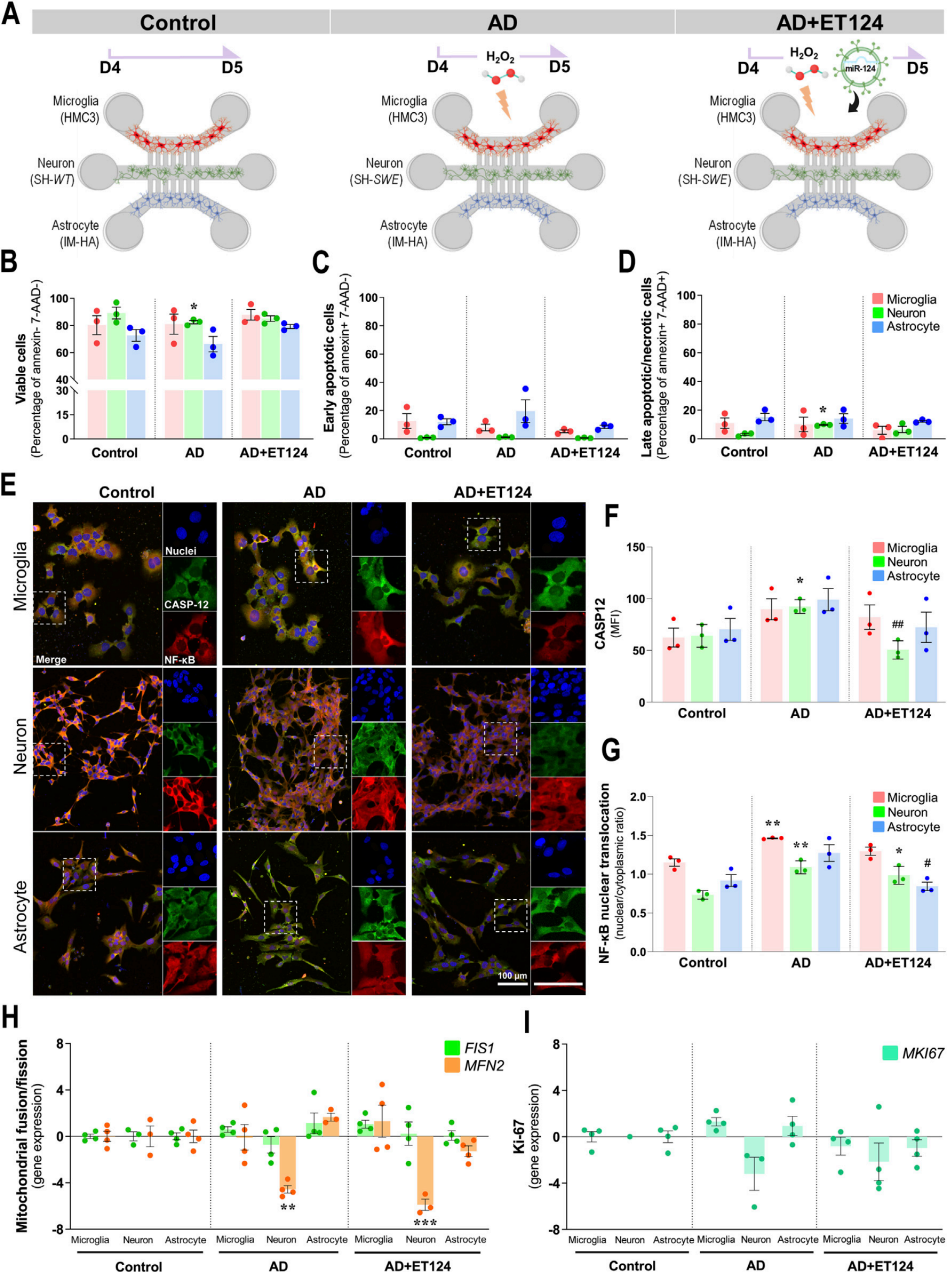
Delivery of ET124 into the AD-like microfluidic triculture model leads to preventive effects on neuronal demise and caspase-12 activation, as well as on neuron-glia NF-κB activation, without affecting mitochondria fragmentation or cell proliferation. **(A)** Schematic representation of ET targeting to control (untreated SH-*WT* cells), AD-like (SH-*SWE* + H_2_O_2_), and AD + ET124 (SH-*SWE* + H_2_O_2_ + ET124) tricultures (microglia–neurons–astrocytes). Evaluation of cell viability was as described in Materials and methods by flow cytometry. Three populations were distinguished: **(B)** Viable (annexin and 7-AAD double-negative); **(C)** early apoptotic (annexin-positive and 7-AAD-negative); and **(D)** late apoptotic (annexin and 7-AAD double-positive). **(E)** Representative immunofluorescence images of caspase-12 (green); NF-κB (red); cell nuclei stained with DAPI (blue) in neurons, microglia, and astrocytes in the microfluidic system tricultures. **(F)** Pixel-integrated measurement of caspase-12 mean fluorescence intensities (MFI, arbitrary unities) by each cell type and condition. **(G)** NF-κB nuclear translocation ratio, obtained by dividing pixel-integrated nuclear/cytoplasmic NF-κB immunofluorescence intensities. Transcription analysis of FIS1 and MFN2 mitochondrial genes **(H)**, and of MKI67, coding for Ki-67 **(I)**, by RT-qPCR. All evaluations were performed in Control, AD, and AD treated with ET124 (AD + ET124). Data are mean ±SEM, from at least three independent experiments. ^***^ *p <* 0.001 vs. Control and ^##^ *p <* 0.01 vs. matched cells in the AD-like model by one-way ANOVA with Tukey's *post hoc* test. SH-*WT, wild-type* human SH-SY5Y cells; SH-*SWE*, SH-SY5Y cells expressing the APP *Swedish* variant; ET124, exosomes isolated from the SH-*WT* cell secretome after transfection with miR-124-3p mimic; IM-HA, immortalized human astrocytes; microglia (human CHME3 cell line); 7-AAD, 7-aminoactinomycin D; CASP12, caspase-12; NF-κB, nuclear factor kappa of activated B cells; *FIS1*, fission 1 mitochondrial gene; *MFN2*, mitofusin2 mitochondrial gene; *MKI67*, Ki-67-coding gene.

As previously stated, our AD-like model is expected to be prone to neuroinflammation and neurodegeneration, leading to cell death. We already mentioned that the SH-*SWE* cells evidenced an increased loss of total neurite length upon the addition of H_2_O_2_, as compared to matched SH-*WT* cells (Supplementary Figure S3), suggesting increased neurotoxicity and ultimately cell demise. Here, we first evaluated how differently the neural cells behaved upon the addition of H_2_O_2_ (Control: SH-*WT* + HMC3 + IN-HA; AD: SH-*SWE* + HMC3 + IN-HA) and whether the addition of ET124 (AD+124) was effective in preventing the loss of cell viability, following the experiments schematized in Figure 4A. Results indicated a subtle but significant neuronal cell demise (*p <* 0.05, Figure 4B) in the AD-like model (SH-*SWE* cells + H_2_O_2_), without early apoptotic alterations (Figure 4C), but an increased percentage of late apoptotic/necrotic cells (*p <* 0.05, Figure 4D). Remarkably, this neurotoxicity was prevented in ET124-treated neurons. No changes were observed in the glial cells, although AD astrocytes (astrocytes in the presence of H_2_O_2_ and neuron-derived pathological signalling) revealed a slight lower viability (with a little more elevated number of apoptotic and necrotic cells).

Once caspase-12 was shown to mediate apoptosis and to be involved in endoplasmic reticulum-associated oxidative stress and Aβ-induced synaptic toxicity (Nakagawa et al., 2000; Quiroz-Baez et al., 2011), as well as in nuclear factor-kappa B (NF-κB) activation (Chow et al., 2021), we decided to evaluate these two parameters by immunocytochemistry (Figure 4E). Elevation of neuronal caspase-12 in the AD model (*p <* 0.05, Figure 4F) was prevented upon the addition of ET124 (*p <* 0.01). It should be noted that caspase-12 showed a trend to increase in astrocytes and microglia in AD and to be counteracted by ET124. NF-κB nuclear translocation was also enhanced in AD neurons and microglia relatively to control (*p <* 0.01, Figure 4G), but ET124 had no power to abrogate such effect, although the significance was reduced to *p <* 0.05 in AD + ET124 neurons. Specifically, ET124 prevented astrocytic NF-κB activation (p < 0.05 vs. AD astrocytes), sustaining control levels. Because NF-κB activation increases mitochondrial fragmentation (Albensi, 2019), we next evaluated *FIS1* and *MFN2* gene expression (encoding for dynamics-associated proteins involved in the mitochondrial fission and fusion mechanisms, respectively) (Kornfeld et al., 2018; Colpman et al., 2023). Our data clearly indicate a significant decrease in neuronal *MFN2* expression (*p <* 0.01) in the AD triculture not solved by the ET124 treatment (Figure 4H), suggesting mitochondria fragmentation. No other significant changes were observed in *FIS1* and *MFN2* gene expression levels across any cell type or condition. Since mitochondrial fusion and fission are involved in the regulation of cell proliferation (Dong et al., 2022), we next assessed the expression of the *MKI67* gene, which encodes the proliferation-associated protein Ki-67 (Figure 4I). However, no significant changes were detected, although neurons tended to exhibit lower levels of *MKI67* compared to microglia and astrocytes. These first data indicate that ET124 exert neuron–glia modulatory effects with promising applications in cell homoeostasis restoring.

### 3.5 Disease-associated alterations in microglia morphometric parameters and phenotypes are modulated by treating the AD-like tricultures with ET124

Data above indicated the presence of microglial activation in the age-AD neural triculture model, as suggested by the increased NF-κB nuclear translocation. In this section, we determined morphological, transcriptional, and immune-associated miRNA microglial changes, known as being associated with AD pathology (Brites, 2020; Franco-Bocanegra et al., 2021; Prater et al., 2023), to better explore the immune-regulatory effects of ET124.

**FIGURE 5:**
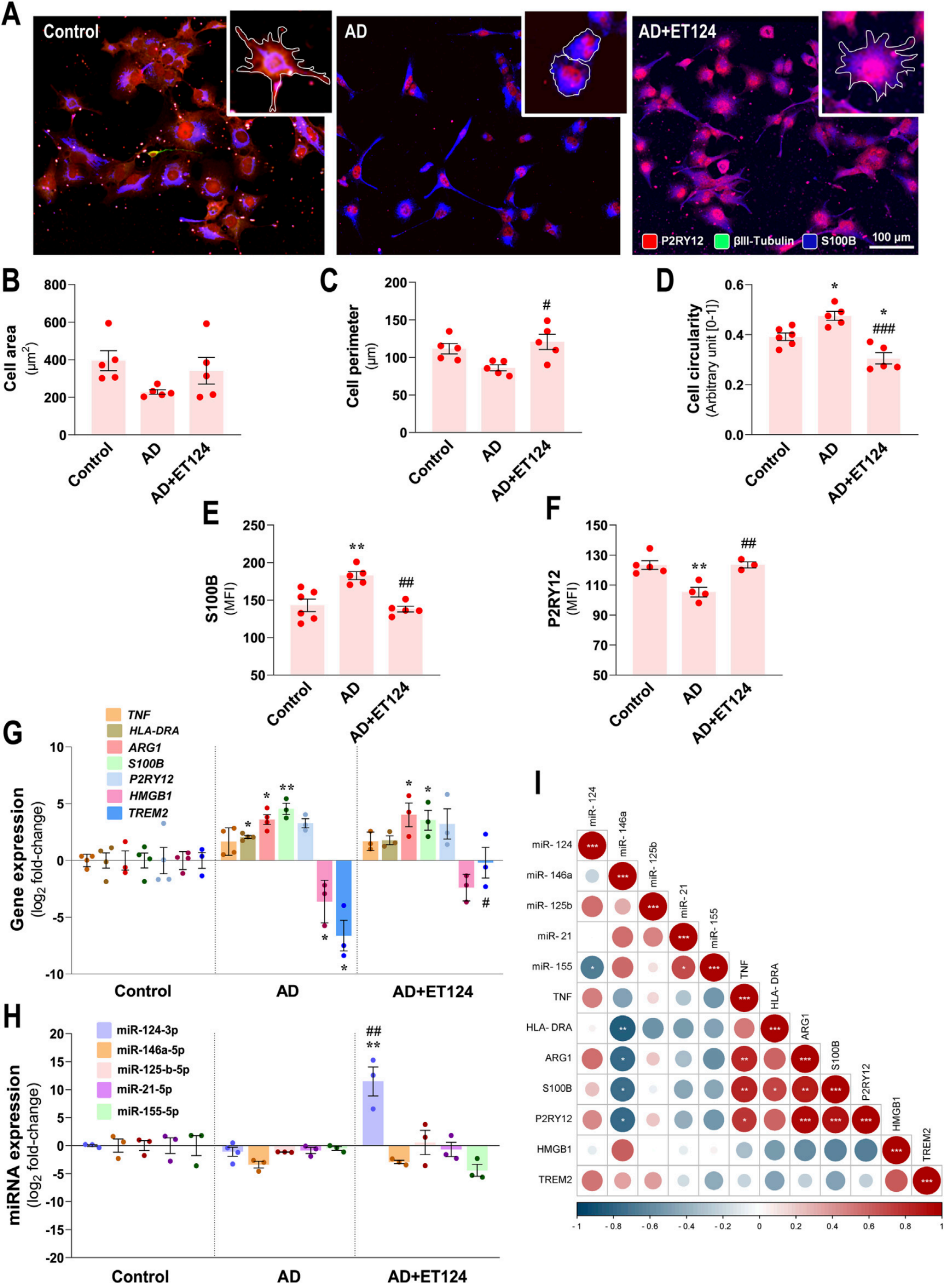
Phenotypic profile of microglia in the AD triculture (microglia–neurons–astrocytes) and consequences by ET124 treatment. **(A)** Representative fluorescence images of P2RY12, βIII tubulin, and S100B immunostaining in the microglial compartment showing P2RY12 specificity to HMC3 microglia with image top-right corner insets (with ×2.25 additional magnification), displaying the most representative cell morphological changes in each condition (white outline). Pixel-integrated fluorescence measurement of cell area **(B)**, cell perimeter **(C)**, and cell circularity **(D)** in each condition. Pixel-integrated mean fluorescence intensities (MFI, arbitrary units) for S100B **(E)** and P2RY12 **(F)**. **(G)** Transcriptional analysis of microglia-associated genes *TNF, HLA-DRA, ARG1, S100B, P2RY12, HMGB1*, and *TREM2*. **(H)** Evaluation of microglial expression of miR-124-3p, miR-146a-5p, miR-125b-5p, miR-21-5p, and miR-155-5p. Gene and miRNA expression levels were evaluated in control (untreated SH-WT cells, *wild-type* human SH-SY5Y cells), AD (SH-*SWE*, SH-SY5Y cells expressing the APP *Swedish* variant + H_2_O_2_), and AD + ET124 conditions by RT-qPCR. Actin (for genes) and U6 (for miRNAs) were used as internal references. **(I)** Correlation matrix based on Pearson’s bivariate coefficients (R2) for pairwise comparisons of microglia-expressed miRNAs and genes. Negative correlations are shown in blue and positive correlations in red, both integrating a respective annotation whenever significant. Data are mean ±SEM, from at least three independent experiments. ^***^ *p <* 0.001 and ^**^ *p <* 0.01 vs. Control; ^###^ *p <* 0.001, ^##^ *p <* 0.01, and ^#^ *p <* 0.05 vs. AD, by one-way ANOVA with Tukey's *post hoc* test. ET124, exosomes isolated from the SH-*WT* cell secretome after transfection with miR-124-3p mimic; astrocytes, IM-HA or immortalized human astrocytes; microglia (human CHME3 cell line); *ARG1*, arginase-1 coding gene; *HLA-DRA*, major histocompatibility complex class II-coding gene; *HMGB1*, high mobility group box protein 1-coding gene; *P2RY12*, purinergic receptor P2Y12-coding gene; *S100B*, S100 calcium-binding protein B; *TNF*, tumour necrosis factor alpha-coding gene; and *TREM2*, triggering receptor expressed on myeloid cells 2 coding gene.

Pixel-integrated image analysis in the age-associated AD model revealed morphological alterations in microglia, compatible with cell polarization (increased number of bipolar and round cells), when compared to control microglia, revealing ramifications (visualized by the white outline). It should be noted that the administration of ET124 to the AD compartment sustained the microglia ramifications and prevented the occurrence of the amoeboid shape (Figure 5A and insets). Although not statistically significant, we observed that microglia in the AD-associated ageing model showed both a reduced area (Figure 5B) and cell perimeter (Figure 5C, *p =* 0.08) that accounted to their significant increase in circularity/roundness shape (*p <* 0.05 vs. control, Figure 5D). Such morphology occurs when microglia switch to an activated phenotype with amoeboid and elongated rod bipolar morphotypes. Targeting of microglia with ET124 produced well-known beneficial effects in counteracting cell perimeter decrease (*p <* 0.05, vs. AD) and circularity increase (*p <* 0.05 vs. control and *p <* 0.001 vs. AD) in the AD condition, including a trend to also sustain cell area control values.

We next assessed S100B and P2RY12, which demonstrated to be upregulated and downregulated, respectively, in pro-inflammatory conditions (Gomez Morillas et al., 2021; Michetti et al., 2023). We observed a significant S100B increase, together with a P2RY12 decrease (*p <* 0.01 for both vs. control, Figures 5E, F), suggesting an activated dysfunctional microglia phenotype. This subtype was validated at the transcriptional level (Figure 5G) by S100B overexpression (*p <* 0.01 vs. control), although the elevation of P2RY12 did not reach significance. ET124 treatment avoided such altered immunoreactivity of S100B and P2RY12 (*p* < 0.01 for both vs. AD). However, no major significant effects were observed at transcriptional levels, despite the lower significance of S100B mRNA vs. control by the delivery of ET124 (*p <* 0.05 instead of the *p <* 0.01 in AD).

Among the microglia gene expression panel (Figure 5G), we detected upregulated *HLA-DRA* (that encodes the major histocompatibility complex class II, *p <* 0.05 vs. control), *ARG1* (*p <* 0.05 vs. control), and *S100B* (*p <* 0.01 vs. control), together with a nearly significant *TNF*α increase (*p =* 0.07 vs. control). We also found a microglial downregulation of *High Mobility Group Box protein 1* (*HMGB1*) and *TREM2* (*p <* 0.05 for both vs. control). All these changes in the AD model suggest the presence of heterogeneous microglial subtypes linked to cell adaptive immune-activation phenotypes. Beneficial outcomes from ET124 addition to the AD triculture were noticed on the sustained expression of *TREM2* near the control levels (*p <* 0.05 vs. AD) and on a slight effect on *HLA-DRA* and *HMGB1* mRNAs (*p =* 0.07 and *p* = 0.1 vs. control, respectively).

Regarding the miRNA profile, no significant miRNA alterations were detected in microglia from the AD system, despite the near-significant miR-146a decrease (*p* = 0.05 vs. control, Figure 5H), persisting after the addition of ET124 (*p =* 0.09). Interestingly, miR-146a levels were shown to inversely correlate with *HLA-DRA* (*p < 0.01*), as well as with *ARG1, S100B*, and *P2RY12* transcriptional levels (*p <* 0.05, Figure 5I), indicating that miR-146a may play a role in conditioning the microglial phenotype. Positive relationships were obtained between *TNF* and *ARG1/S100B/P2RY12* genes, as well as between *S100B* and *HLA-DRA* (at least *p <* 0.05), confirming their relevance in categorizing microglial subtypes. A more prominent effect was observed for the upregulation of miR-124-3p by ET124 (*p <* 0.01 vs. control and vs. AD) as we anticipated considering the higher ET124 delivery into microglia, which may account for the non-statistical decrease of miR-155-5p (*p* = 0.10 vs. AD). Such antagonism between microglial miR-124-3p and miR-155-5p was manifested by a significant negative correlation between both miRNAs (*p <* 0.05, Figure 5I). Together, these data suggest a more functional and homoeostatic microglia phenotype in the AD + ET124 model.

### 3.6 ET124 mitigates H_2_O_2_-induced neurite atrophy and prevents *NOS1*, *S100B*, and miR-146a AD-associated dysregulation

**FIGURE 6:**
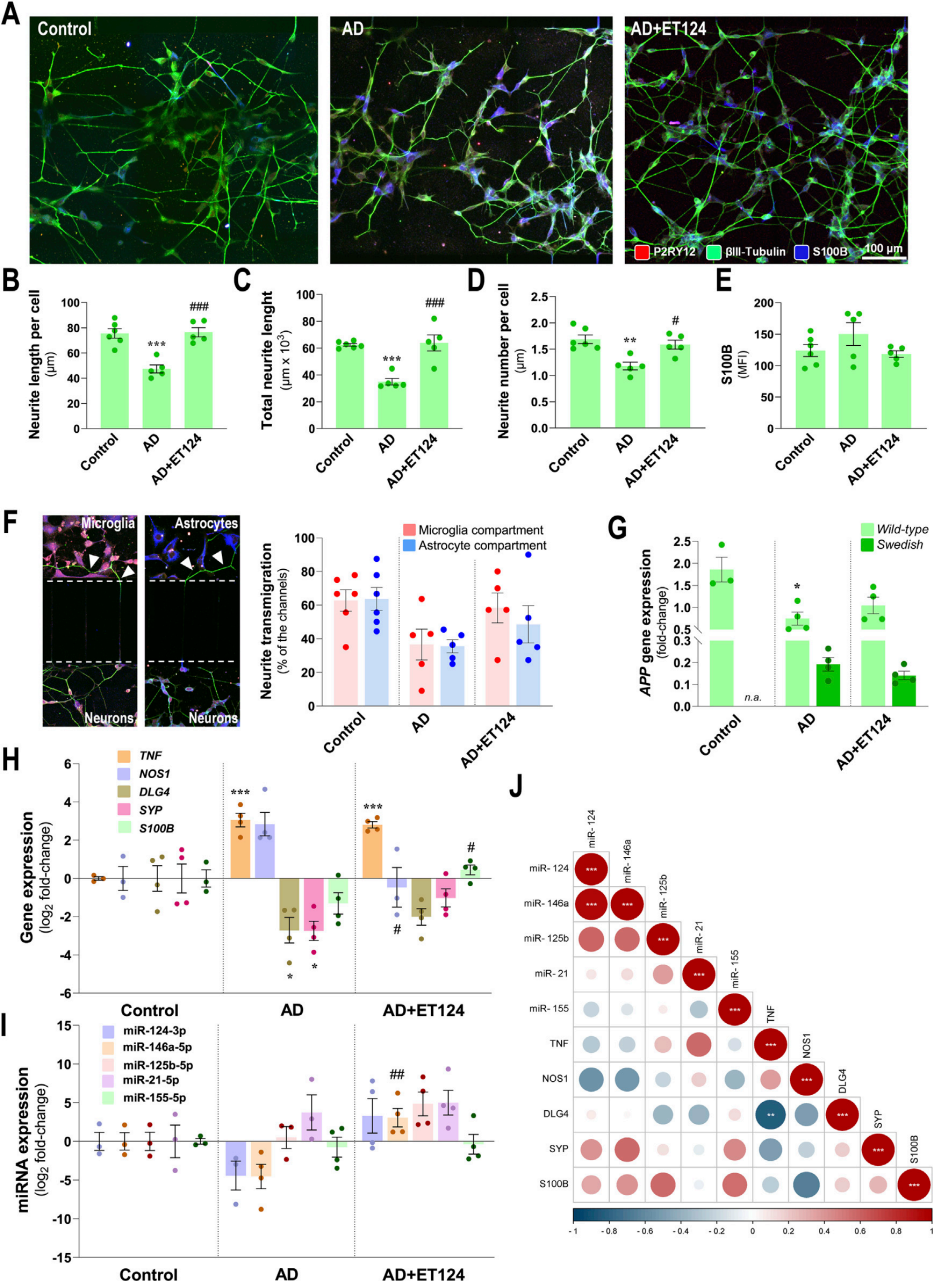
Characterization of the neuronal pathological features in the AD triculture (microglia–neurons–astrocytes) system and of the preventive effects by ET124 treatment. **(A)** Representative fluorescence images of P2RY12, βIII tubulin, and S100B immunostaining in the neuronal compartment, showing βIII tubulin specificity to neuronal cells/neurites in the three experimental conditions: SH-SY5Y neurons in non-treated tricultures (Control); SH-*SWE* in AD model tricultures treated with H_2_O_2_ (AD); and SH-*SWE* in AD model tricultures treated with ET124 *(*AD + ET124). Pixel-integrated fluorescence measurement of neurite length per cell **(B)**, total neurite length **(C)**, and neurite number per cell **(D)**. Pixel-integrated mean fluorescence intensities (MFI, arbitrary units) for S100B **(E)**. **(F)** Representative fluorescence image sections of the transmigrating neurites (neurites interacting with microglia and/or astrocyte compartments) through the silicon-separating block, accompanied by the percentage of transmigrating neurites counted in microglia and astrocyte compartments. **(G)** Transcriptional level of amyloid precursor protein (APP) using primers that discriminate *WT* and *SWE* forms by RT-qPCR. **(H)** Gene expression analysis of neuronal-associated TNF, NOS1, DLG4, SYP, and S100B. **(I)** Expression levels of the neuronal miR-124-3p, miR-146a-5p, miR-125b-5p, miR-21-5p, and miR-155-5p. Gene and miRNA expression levels were evaluated in Control (untreated SH-WT cells, *wild-type* human SH-SY5Y cells), AD (SH-*SWE*, SH-SY5Y cells expressing the APP *Swedish* variant + H_2_O_2_), and AD + ET124 conditions by RT-qPCR. **(J)** Correlation matrix based on Pearson’s bivariate coefficients (R^2^) for pairwise comparisons of neuron-expressed miRNAs and genes. Negative correlations are shown in blue, and positive correlations, in red, both integrating a respective annotation whenever significant. Data are mean ±SEM, from at least three independent experiments. ^***^ *p <* 0.001, ^**^ *p* < 0.01, and ^*^ *p* < 0.05 vs. Control; ^###^ *p <* 0.001, ^##^ *p* < 0.01, and ^#^ *p <* 0.05 vs. AD, by one-way ANOVA with Tukey's *post hoc* test. ET124, exosomes isolated from the SH-*WT* cell secretome after transfection with miR-124-3p mimic. Astrocytes, IM-HA or immortalized human astrocytes; microglia (human CHME3 cell line); NOS1, neuronal nitric oxide synthase-coding gene; DLG4, postsynaptic density protein 95 (PSD95)-coding gene; S100B, S100 calcium-binding protein B-coding gene; SYP, synaptophysin-coding gene; TNF, tumour necrosis factor alpha-coding gene.

In this section, we focused on changes occurring preferentially in the neuronal compartment. As commented above, relatively to the increased loss of total neurite length upon the addition of H_2_O_2_, as compared to matched SH-*WT* cells in monocultures (Supplementary Figure S3), the same was observed in our AD tricultures, as revealed by projections identified with βIII-tubulin immunostaining (*p <* 0.001 vs. control, Figures 6A, B). Changes also included the reduction in total neurite length (*p <* 0.001 vs. control, Figure 6C) and neurite number per cell (*p <* 0.01 vs. control, Figure 6D). Marked preventive effects were observed upon ET124 treatment, for neurite length per cell and total neurite length (*p <* 0.001 vs. AD) with values like those of control, as well as for the neurite number per cell (*p <* 0.05 vs. AD).

Neuronal S100B is considered a reliable marker of active neural distress (Michetti et al., 2023). Although a slight elevation was identified in neuronal S100B immunofluorescence intensity in the AD system, no significant changes were observed for any tested condition (Figure 6E). Relatively to the number of transmigrating neurites (neurites that migrated through the microchannels towards microglial and astrocytic compartments) (Figure 6F), data suggest a reduced transmigration to the microglial and astrocyte compartments (*p* = 0.09 and *p* = 0.05, vs. control, respectively) in the age-AD model, as well as a protective effect by ET124 treatment, especially in the microglial compartment where values closer to control levels were observed.

By using specific discriminating primers, we confirmed that SH-*SWE* cells from the AD system express both *WT* and *SWE* transcript variants of the *APP* gene, unlike SH-*WT* used in the control system (Figure 6G). Nevertheless, a significant APP-WT downregulation (*p <* 0.05 vs. control) was detected in the AD system but not upon ET124 treatment (*p* = 0.05 vs. control). No changes were detected on APP-SWE expression, regardless of the ET124 treatment. Regarding other transcripts, the SH-*SWE* neurons from the AD triculture evidenced increased levels of *TNF* (*p <* 0.001 vs. control) and *NOS1* (*p* = 0.07 vs. control), together with decreased expression of *DLG4* (postsynaptic density protein 95 (PSD95)-coding gene) and *SYP* (*p <* 0.05 vs. control, for both), suggesting the presence of neuroinflammation and impaired synaptic dynamics (Figure 6H). Treatment with ET124 did not counteract *TNF* but led to a significant decrease in *NOS1* (*p <* 0.05 vs. AD) and to less defective *DLG4* and *SYP* mRNAs, together with a small significant increase in S100B (*p <* 0.05 vs. AD), suggested to be an Aβ42 suppressor (Cristóvão et al., 2018).

In what concerns the inflammation-associated miRNA profile (Figure 6I), no significant alterations were observed in the neurons from the AD triculture, as compared to the control condition. However, treatment with ET124 led to miR-146a upregulation (*p <* 0.01 vs. AD), as well as to moderate elevation of miR-124 (*p* = 0.06 vs. AD), not surprisingly if we consider that neurons are not the cells that most engulf ET124. When looking for correlations among microglial genes and miRNAs, we found a strong positive correlation of miR-124-3p with miR-146a-5p (*p <* 0.001) and a negative correlation between the expression of *TNF* and *DLG4* genes (*p <* 0.01), as shown in Figure 6J.

Altogether, these results support a neuroprotective effect of ET124 over the neuropathological features exhibited by the AD neuronal compartment in the microfluidic tricultures.

### 3.7 ET124 halts astrocyte morphological and immune dysregulation in the AD triculture model

Astrocytes, as the most abundant cell type in the CNS, show deleterious effects in AD, exacerbating Tau hyperphosphorylation and Aβ pathology (Cai et al., 2017), and acquiring several pathological phenotypes with altered morphologies and exacerbated reactivity (Kim et al., 2024). Here, morphological, transcriptional, and immune-associated miRNA astrocyte changes were investigated in the AD triculture model, similarly to that performed for microglia, together with the assessment on whether ET124 exerted a positive outcome on astrocyte-induced neuroinflammation.

**FIGURE 7:**
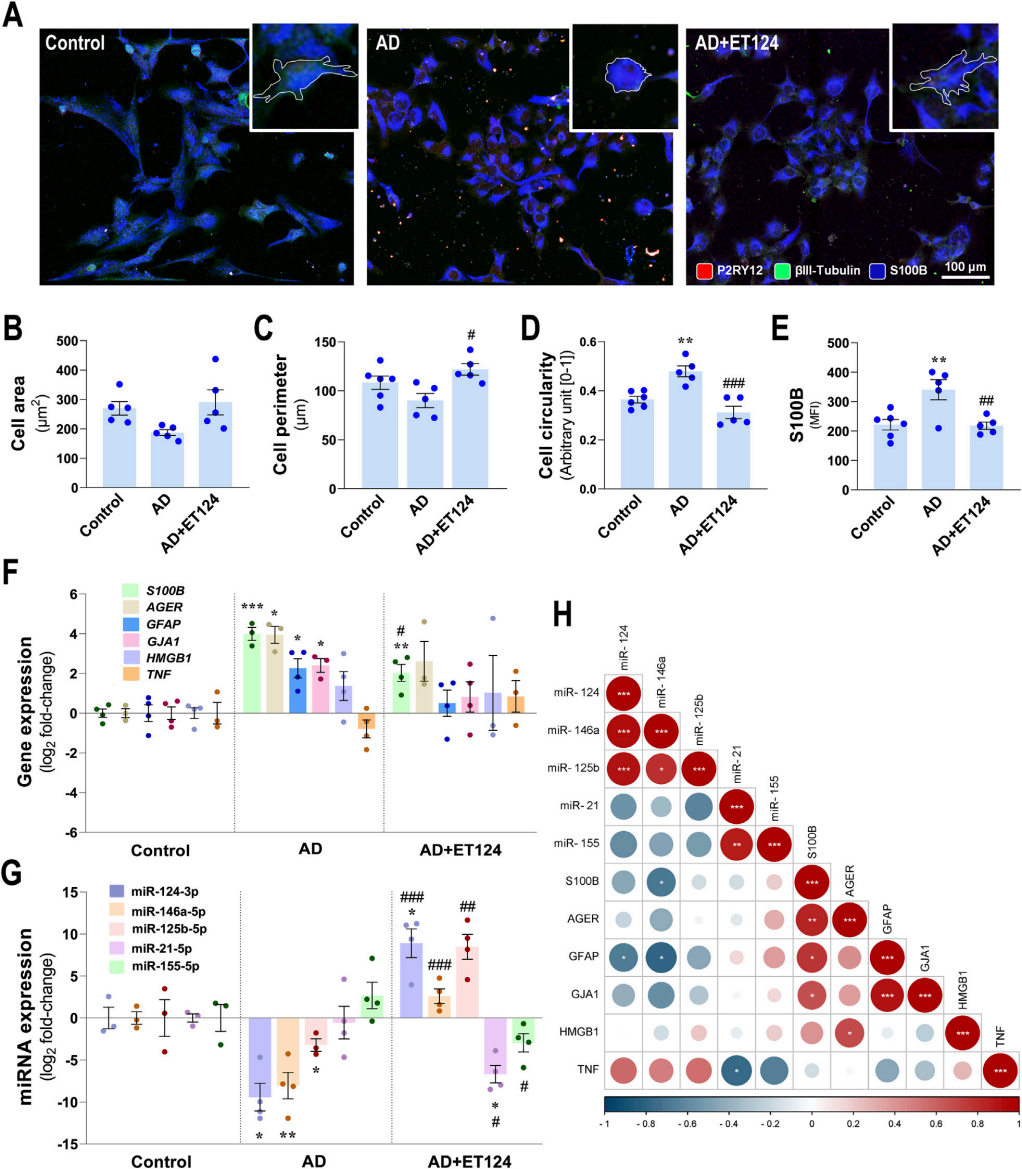
Phenotypic profile of astrocytes in the AD triculture (microglia–neurons–astrocytes) and benefits by ET124 treatment. **(A)** Representative fluorescence images of P2RY12, βIII tubulin, and preferential S100B immunostaining in the astrocyte compartment with image top-right corner insets (with ×2.25 additional magnification), displaying the most representative cell morphological changes in each condition (white outline). Pixel-integrated fluorescence measurement of the cell area **(B)**, cell perimeter **(C)**, and cell circularity **(D)** in each condition. Pixel-integrated mean fluorescence intensities (MFI, arbitrary units) for S100B **(E)**. **(F)** Transcriptional analysis of the astrocyte-associated genes *S100B, AGER, GFAP, GJA1, HMGB1,* and *TNF*. **(G)** Evaluation of astrocytic expression of miR-124-3p, miR-146a-5p, miR-125b-5p, miR-21-5p, and miR-155-5p. Gene and miRNA expression levels were evaluated in Control (untreated SH-WT cells, *wild-type* human SH-SY5Y cells), AD (SH-*SWE*, SH-SY5Y cells expressing the APP *Swedish* variant + H_2_O_2_), and AD + ET124 conditions, by RT-qPCR. Actin (for genes) and U6 (for miRNAs) were used as internal references. **(H)** Correlation matrix based on Pearson’s bivariate coefficients (R^2^) for pairwise comparisons of astrocyte-expressed miRNAs and genes. Negative correlations are shown in blue, and positive correlations, in red, both integrating a respective annotation whenever significant. Data are mean ±SEM, from at least three independent experiments. ^***^ *p <* 0.001, ^**^ *p <* 0.01, ^*^ *p <* 0.05 vs. Control; ^###^ *p <* 0.001, ^##^ *p <* 0.01, and ^#^ *p <* 0.05 vs. AD, by one-way ANOVA with Tukey's *post hoc* test. ET124, exosomes isolated from the SH-*WT* cell secretome after transfection with miR-124-3p mimic. Astrocytes, IM-HA or immortalized human astrocytes; microglia (human CHME3 cell line); *AGER*, receptor for advanced glycation end product-coding gene; *GFAP*, glial fibrillary acidic protein coding gene; *GJA1,* gap junction protein alpha 1 gene (coding for connexin 43); *HMGB1*, high mobility group box protein 1-coding gene; *S100B*, S100 calcium-binding protein B-coding gene; *TNF*, tumour necrosis factor alpha-coding gene.

As evidenced in Figure 7A, IM-HA cells with predominant S100B immunostaining showed bushy, spongy, and star-like morphologies in the control condition. Morphological deficits (decreased branching) were present predominantly in the AD-like system. Such a change is better visualized when comparing astrocyte morphologies in the insets (defined by the white outline). Astrocyte processes are visible in the control and ET124 experiments, indicating the efficacy of this treatment to sustain their native morphology. Morphometric studies revealed a non-significant reduction in the cell area (Figure 7B) and perimeter (Figure 7C) but an enhanced cell circularity in the age-AD model (*p <* 0.01 vs. control, Figure 7D), like those previously found for microglia in the same condition. ET124 treatment showed to prevent such morphological alterations by sustaining the cell area at control levels (p = 0.06 vs. AD) but mainly by preventing changes in the cell perimeter (*p <* 0.01 vs. AD) and circularity (*p <* 0.001 vs. AD). These regulatory effects of ET124 on astrocyte morphology and similarity to that observed for the microglial cells indicate that the targeting of ET124 equally prevents such a glial cell-altered shape in the AD compartment, despite the lower levels of PKH67 exosome internalization when compared to those in microglia (Figure 3G).

Like microglia, and in part neurons, astrocytes from the AD tricultures also exhibited increased S100B immunofluorescence (*p <* 0.01 vs. control, Figure 7E). Again, this reactivity-associated marker was prevented by the ET124 treatment (*p <* 0.01 vs. AD), further adding to its therapeutic potential in mitigating astrocyte-associated pathology in the triculture AD model.

Determination of astrocyte-associated inflammatory genes further validated reactive astrogliosis to the disturbed homoeostasis in the AD-like model (HMC3/IM-HA/SH-*SWE* and H_2_O_2_). Indeed, AD astrocytes showed a significant upregulation of the genes *S100B* (*p <* 0.001 vs. control), *AGER* (encoding the advanced glycosylation end-product-specific receptor, *p <* 0.05 vs. control), *GFAP* (*p <* 0.05 vs. control), and *GJA1* (encoding gap junction protein alpha 1, *p <* 0.05 vs. control). These findings were counteracted by ET124 (Figure 7F) that significantly diminished *S100B* upregulation (*p <* 0.01 vs. AD) and attenuated overexpression of *AGER*, *GFAP*, and *GJA1* genes (though not significantly vs. control or AD models). Data obtained so far validate the immunomodulatory potential of ET124 over the glial activation and neurodegeneration in our AD-like model.

Most important, we found a downregulation of several anti-inflammatory miRNAs in AD-related astrocytes, corroborating the homoeostatic imbalance and neuroimmune dysregulation in this system. We observed a defective expression of miR-124-3p (*p <* 0.05 vs. control), miR-146a-5p (*p <* 0.01 vs. control), and miR-125b-5p (*p* < 0.05 vs. control) (Figure 7G). A rebalancing action has been achieved by targeting the whole system with ET124, as evidenced by the upregulated anti-inflammatory miRNAs (miR-124-3p, *p <* 0.001; miR-146a-5p, *p <* 0.001; and miR-125b-5p, *p <* 0.01, all vs. AD). In addition, ET124 also inhibited the expression of miR-21-5p (*p* < 0.05 vs. control and AD), which regulates multiple AD pathologies, as well as miR-155-5p (*p <* 0.05 vs. AD), usually considered to be eliciting a pro-inflammatory response.

Such dynamic gene expression and miRNA profile led us to conduct Pearson’s correlation analysis that highlighted interesting associations (Figure 7H). For instance, miR-124-3p showed to be positively correlated with miR-146a-5p and miR-125b-5p (*p* < 0.001, for both) and negatively correlated with *GFAP* (*p <* 0.05). Such findings support ET124 potential as a new and efficient therapeutic approach to halt astrocyte-induced AD pathogenesis. miR-146a-5p regulation by ET124 also have positive impact on miR-125b-5p (*p <* 0.05) and negative impact on *S100B* and *GFAP* (*p <* 0.05, for both). miR-21-5p directly correlated with miR-155 (*p <* 0.01) and inversely with *TNF* (*p <* 0.05), suggesting non-synergetic effects on inflammation. Other positive correlations included *HMGB1* with *AGER* (*p <* 0.05); *GJA1* with *GFAP* (*p <* 0.001); and *S100B* with *AGER*, *GFAP*, and *GJA1* (*p <* 0.01, *p <* 0.05, and *p <* 0.05, respectively). It should be additionally noted that intercellular direct correlations for the astrocytic miR-146a-5p and miR-125b-5p with matched miRNAs in neurons, and of miR-124-3p with the microglial one (Supplementary Figure S4), further pointed to miRNA paracrine signalling.

These last data confirm the ET124 potential to counteract astrocyte reactivity in a dynamics neuron–glial system that mimics the AD microenvironment, ultimately supporting their application to modulate inflammation and age-associated neurodegeneration.

## 4 Discussion

AD persists as the most prominent form of dementia worldwide, being estimated to increase in the coming decades (Yiannopoulou and Papageorgiou, 2020). Treatments, such as memantine—an NMDA receptor antagonist that prevents nerve cell damage by excessive glutamate—and donepezil that slows the breakdown of acetylcholine improving mental and behavioural functions, do not inhibit the cognitive decline and death of brain cells (Korczyn and Grinberg, 2024). Lately, anti-Aβ therapy with monoclonal antibodies has shown some favourable effects, but only a small percentage of older adults with early cognitive impairment have been considered eligible (Pittock et al., 2023).

Therefore, better therapeutic approaches, effective biomarkers for identifying patients with preclinical AD, better translational models, and novel tools to more closely mimic AD pathology are required to connect basic and clinical research (Zhang Y. et al., 2023). Such experimental models recapitulating AD pathological hallmarks are crucial for gaining a better knowledge on the underlying mechanisms and for testing innovative therapeutic approaches. Translation of successful results in transgenic mice to clinics has shown a high-rate failure and limitations of the model (Drummond and Wisniewski, 2017). Using human cells as experimental models overcomes species differences.

Lately, miRNAs have been emerging as promising therapeutic molecules for brain diseases based on preclinical studies and clinical trials, although it is not clear their specific turnover mechanisms, feasibility, effectiveness, and safety (Ma and Zhao, 2023). Moreover, one of the key issues of miRNAs in therapeutics is their instability and rapid degradation that impair specificity and leads to off-target effects, as well as their difficulty in crossing biological barriers (Zhang et al., 2021). Exosomes have the advantage of crossing the BBB carrying signalling molecules, proteins, lipids, and non-coding RNAs (e.g., miRNAs) that control gene expression in the recipient cells (Cunha et al., 2016; Loch-Neckel et al., 2022). Several studies identified alterations in the miRNA expression profile in AD, and its modulation has successfully demonstrated neuroprotective effects in different models (Sun et al., 2018; Garcia et al., 2022). Mesenchymal and stem cell-derived exosomes have shown therapeutic potential in AD mouse models, as well as in rodent/human neuronal monoculture AD models (Xiong et al., 2021; Huber et al., 2022). Furthermore, exosomes have been loaded with different miRNAs for therapeutic purposes, such as with miR-146a mimic for immunomodulatory effects in rheumatoid arthritis and spinal cord injury (Tavasolian et al., 2020; Lai et al., 2022) or with the miR-155 inhibitor in oral cancer (Sayyed et al., 2021). Similarly, enriched miR-124-3p exosomes were used for diseases such as Huntington’s [generated from transfected HEK 293 cells (Lee et al., 2017)] and Parkinson’s [generated from human umbilical cord blood-derived mononuclear cells (Esteves et al., 2022)], or even for repetitive mild traumatic injury [generated from BV2 microglial cells (Ge et al., 2020)]. As far as we know, only engineered dendritic cell-derived miR-29b-2-exosomes were tested in diseased SH-SY5Y cells and 3xTg-AD mice, as in AD models (Lin et al., 2024).

Therefore, the present work was pioneer in developing miR-124-3p-loaded neuronal exosomes as a strategy to prevent AD progression and in using human neural tricultures in microfluidic devices, allowing cell-to-cell communication between neuron-like (SH-WT human neuroblastoma cells in control experiments or SH-SWE in the AD system), astrocytes and microglia cell lines (IM-HT and HMC3, respectively).

Like other authors, we used SH-*SWE* cells as an AD model (Joh and Choi, 2017; Fernandes et al., 2018; Garcia et al., 2021; Garcia et al., 2022). The use of the SH-SY5Y neuroblastoma cell line, a cloned subline of a neuroblastoma cell line from a bone marrow biopsy (Feles et al., 2022), has been commonly used, and most of the limitations of the model are associated with the fact that SH-SY5Y cells are undifferentiated (Lopez-Suarez et al., 2022), which was not the case in the present study, due to the differentiation protocol with RA that upregulates neuronal markers and the terminal phenotype (Xicoy et al., 2017). Because they are an immortalized cell line, SH-*SWE* cells may present carcinogenic factors (Maqsood et al., 2013) and genetic peculiarities that may lead to an unexpected response to a determined insult (Lopez-Suarez et al., 2022). Moreover, this and other cell lines do not mimic microenvironment perturbations. However, this aspect was surpassed using a triculture model that allows interactions between neurons and glial cells. Lately, induced pluripotent stem cells (iPSCs) generated from patient fibroblasts and differentiated into neurons were shown to better reproduce sporadic and familiar cases of AD (Poon et al., 2017), but limitations due to difficult recapitulation of cell ageing and phenotypic variation also need to be overcome (Volpato and Webber, 2020; Jothi and Kulka, 2024). Direct reprogramming minimizes safety concerns due to iPSCs, although poor proliferative ability and low efficiency limit its application (Zhang Y. X. et al., 2022). Therefore, we decided to use SH-*WT* and SH-*SWE* cells because they can be maintained in culture for long periods of time, are cost-effective, easy to work, more reproducible, provide more material, and bypass ethical concerns (Garcia et al., 2021). Furthermore, the culture of these cells in our multicompartment microfluidic system coated with poly-D-lysine and laminin allows their expansion, migration, and ramification under more physiologic conditions, if compared to a normal T-flask or Petri dish. Finally, SH-*SWE* cells were shown to release Aβ1-40, one of the hallmarks of AD (Fernandes et al., 2018).

In our previous studies, we identified that exosomes from SH-*SWE* cells have increased levels of miR-124, miR-21, and miR-125b, when compared to those from SH-*WT* cells, which revealed to be internalized by HMC3 microglia (the human microglial clone 3 cell line) after 24 h incubation and to co-localize with lysosomes (Fernandes et al., 2018). These lysosomes revealed to be degraded in the following 24 h. Microglia showed upregulated levels of miR-21, HMGB1, TNF-α, and S100B, thus supporting their activation by SH-*SWE*-derived exosomes. We also observed the existence of miR-124 in hippocampal microglia (*in situ* hybridization) from a Braak stage VI AD patient and homogenates (RT-qPCR) from postmortem biopsies of AD patients (Brites, 2020). In these last specimens from Braak stages II–V/VI AD patients, miR-124 upregulation was only observed in Braak stage III. Interestingly, we additionally identified increased miR-124 levels in the cortex, but not the hippocampus, of 3xTg-mice at 3 months, with downregulated levels at 9 months (Fernandes et al., 2022). It should be noted that miR-124 was shown to have neuroprotective properties in AD pathogenesis by targeting β-site APP cleaving enzyme 1 (BACE1) expression (Fang et al., 2012). Elevation of miR-155 in samples of the transgenic mice vs. WT at both 3- and 9-month-old in the cortex and hippocampus (except at the age of 3 months), together with *TNF-α*, *IL-1β, HMGB1*, and *iNOS* gene expression, mainly in the hippocampus, suggests the presence of neuroinflammation in this 3xTg-mouse model.

Although microglia and astrocytes have revealed regional diversity (Grabert et al., 2016; Batiuk et al., 2020), turning interesting to produce cortical and hippocampal regional tricultures, we faced the impossibility to obtain iPSC-derived regional microglial cultures and the limitation that only iPSC-derived cortical astrocytes are described in the literature (Hedegaard et al., 2020). Despite the possibility of modern genetic and cell lineage tracing tools, high-throughput sorting, and high-resolution sequencing technologies (Tan et al., 2020), such issues make it currently unfeasible to carry out regional human tricultures. With this in mind, we decided to proceed with human immortalized HCME3 microglia and human astrocyte (IM-HA) cell lines for tricultures with the SH-*SWE* and SH-*WT* human neuroblastoma cells.

Concerning the HMC3 cell line, established through SV40-dependent immortalization of human embryonic microglial cells, cells revealed surface markers, phagocytic properties, and inflammatory responses upon pro-inflammatory stimuli like primary microglia (https://bitesizebio.com/48560/microglial-cell-lines/). It should be noted, however, that the cells in homeostatic conditions were shown to produce significant amounts of reactive oxygen species (ROS) and IL-6 (Dello Russo et al., 2018). HMC3 cells present low-to-absent expression of CD14 and CD11b, like the human iPSC-derived microglia, and positivity for IBA1, Cx3CR1, TMEM119, P2RY12, and TREM2, which validates the model (Fazzina et al., 2024). To mention that when microglia are removed from the brain and placed into a plastic dish, in monoculture, they stop receiving brain-specific signals, such as the release of chemokines by neurons. However, in our triculture model, they were receiving signals from neurons and astrocytes about disturbances on their health, which turns the system a better recapitulation of the *in vivo* system. In addition, the use of H_2_O_2_ in low dose provided a better approximation to the physiologic oxidative exposure that these cells experiment *in vivo*, when ageing, and surpassed the lack of age-related features typical of immortalized cell lines.

Considering the IM-HA cells, they were established from primary cultures of human cortical astrocytes with a SV40 large T-antigen (https://cells-online.com/product/immortalized-human-astrocytes/). They are supplied as purified astrocytes, produce faster than primary cultures, and grow for extended periods of time. As limitations, they express reduced levels of GFAP but not of S100B and may behave phenotypically like reactive astrocytes (without oligodendrocyte-type 2 astrocyte progenitors’ cells but with vimentin and nestin) (Morita et al., 2021). Similar to all immortalized cells, they differ from “normal” cells because they may express unique gene patterns. Furthermore, when the number of passages increases, immortalized cells may accumulate epigenetic changes (Voloshin et al., 2023). In our study, we only utilized astrocytes in early passages.

Tricultures were delivered into a tricompartmentalized microfluidic system, previously developed (De Vitis et al., 2021), which overcome the low yield of exosomes when generated from monocultures and have the advantage over the coculture mixed system (Park et al., 2018) to facilitate independent microglia–neuron–astrocyte signalling. This tool was the key to assess the effects of ET124 in each human cell type of the AD system. Neurons, microglia, and astrocytes were simultaneously stressed with H_2_O_2_ (in AD system) or co-treated with H_2_O_2_ + ET124 (in AD + ET124 system), while controls did not receive any treatment. The addition of H_2_O_2_ intended to stimulate ageing-associated disease features (Chadwick et al., 2010; Ismail et al., 2016). The model not only recapitulated AD pathological ageing features but also permitted to test ET124 in minimal amounts.

Our decision to upregulate miR-124 in the triculture microfluidic system derived from our previous experiments, where it showed neuroprotective properties (Garcia et al., 2021; Garcia et al., 2022) but also because miR-124 is the most predominant miRNA in the adult brain (Zhang W. H. et al., 2023). It is mostly expressed by neuronal cells and engaged in a variety of biological processes, including neuronal development and differentiation, synaptic plasticity, neurite outgrowth control, and even the acquisition and maintenance of neuronal identity (Yu et al., 2008; Sun et al., 2015). Using experimental *in vitro* models, we demonstrated that miR-124 inhibition in neurons affects the dendritic spine number and increases *APP* gene expression (Garcia et al., 2021). In contrast, in neurons differentiated from AD patient-derived iPSCs, miR-124 overexpression not only prevented *APP* overexpression and oligomerization of toxic amyloid species but also reduced tau phosphorylation and preserved the dendritic spine number (Garcia et al., 2021). Therefore, our ET124 strategy may reveal particularly interesting in patients showing miR-124 downregulation, a condition previously found in hippocampal biopsy samples from AD individuals (Lukiw, 2007; Smith et al., 2011).

Additionally, miR-124-3p also acts through the inhibition of pro-apoptotic factors and by promotion of cellular survival pathways. For instance, miR-124 interacts with the STAT3 pathway, which is crucial for reducing oxidative stress and maintaining mitochondrial function (Geng et al., 2024). We previously demonstrated that neuronal overexpression of miR-124 also exerted a strong paracrine effect over cocultured IFNγ-stimulated microglia, by redirecting their proteomic profile towards a pro-regenerative and anti-inflammatory phenotype, in accordance with other studies (Ponomarev et al., 2011; Garcia et al., 2022). By proteomic analysis, we identified multiple Wilms tumor suppressor gene (WT1) regulators as differentially expressed according to miR-124-3p levels, including the PRKC apoptosis WT1 regulator (PAWR). Since 2011, several authors have demonstrated that miR-124 acts as a powerful immune modulator, suppressing pro-inflammatory microglial responses and inducing anti-inflammatory gene expression via targeting CCAAT/enhancer-binding protein-α (C/EBP-α) (Ponomarev et al., 2011; Yu et al., 2017; Veremeyko et al., 2019). Additionally, miR-124 has been shown to mitigate microglial activation triggered by surgical trauma (Chen et al., 2019) and to mediate morphine-induced inhibition of innate immunity in microglia (Qiu et al., 2015). Furthermore, miR-124 combined with bone marrow-derived exosomes has been shown to inhibit the p38 MAPK signalling pathway, leading to the upregulation of the glutamate transporter GLT-1, mitigating neural apoptosis (Zhuang et al., 2023). Although miR-124 has been poorly explored in AD, there are consistent lines of evidence, supporting its critical role in APP transcription and alternative splicing (Smith et al., 2011; Garcia et al., 2021), BACE1 expression (Fang et al., 2012; An et al., 2017), and tau hyperphosphorylation (Kang et al., 2017; Hou et al., 2020).

In summary, the regulation/modulation of miR-124 was envisaged, in this study, as a potential therapeutic strategy for AD, and the primary endpoint was to develop an exosome-based strategy for miR-124-3p delivery. For this, we first transfected miR-124 in SH-*WT* cells, followed by the isolation of miR-124-loaded exosomes (CT124), as other authors did with HEK 293 cells (Lee et al., 2017). Alternatively, we directly transfected SH-*SW*-exosomes with miR-124 (ET124), using Exo-Fect (de Abreu et al., 2021; Esteves et al., 2022). Although ET124 revealed to be the best approach in terms of transfection efficiency, both ET124 and CT124 were tested as cell targeting tools in 2DIV stressed cortical microglia. These microglia isolated from 2-day-old mice, cultured for 21 days in mixed culture with paired astrocytes, and then isolated and cultured for 2DIV revealed a pro-inflammatory phenotype when compared with the stabilized 10DIV microglia (Caldeira et al., 2014). In this condition, we could realize that both CT124 and ET124 modalities succeeded in raising microglial miR-124-3p expression levels, although ET124 was 20-fold more efficiently delivered than CT124. This finding is in line with studies from Abreu and colleagues, when several miR-155 loading processes were used and direct exosome transfection with Exo-Fect was shown as the best procedure (de Abreu et al., 2021). Our finding on increased ET124 internalization vs. CT124 may later deserve membrane lipidomic studies and elucidation of the engulfment process by electron microscopy.

Microglial phenotypes are governed by the surrounding environment and may show different coexistent phenotypes in neurodegenerative diseases, such as AD (Moore et al., 2015; Prater et al., 2023). Microglial upregulation of *Nos2* gene (encodes for iNOS) upon treatment with both the mock and CT124 exosomes but not with ET124 suggests a selective response to those exosome preparations, impacting on the generation of ROS and inflammation (Sierra et al., 2014). On the other hand, the higher *Arg1* upregulation by ET124 vs. CT124 indicates their increased anti-inflammatory potential, once Arg1 showed to antagonize iNOS in myeloid cells (Shosha et al., 2023). In the same way, *Trem2* upregulation by ET124 vs. CT124 is in line with an enhancement of microglial phagocytosis (Akhter et al., 2021). TREM2 was shown to be fundamental for promoting microglial activation in Aβ and tau pathologies and considered a target to restore homoeostatic microglia (Qin et al., 2021). These data favour ET124 over CT124 in supporting a neuroregenerative microglia subtype.

Co-expression patterns of microglia markers Iba1 and P2Ry12 were found in AD, and phenotypes Iba1^high^ and P2RY12^low^ were identified around Aβ plaques associated with cell activation and impaired phagocytosis. Microglia showing P2RY12^high^ are associated with the homeostatic state, having motility and migration potential abilities, while also occurring in early stages of activation and far from Aβ plaques (Gomez Morillas et al., 2021; Kenkhuis et al., 2022). Specifically, all incubations with exosomes attenuated *Iba1*, despite the strongest efficiency of ET124. The profile was the inverse for the *P2ry12* expression, where the decrease was only produced by CT124. By upregulating microglial *P2ry12* levels, ET124 further validated their promise as a therapeutic strategy.

Neurons are implicated in cognitive functions mediated by a complex and dynamic circuitry based on synaptic communication (Pan and Monje, 2020). Microglia and astrocytes coordinate each other and orchestrate most of the brain’s inflammatory properties, with a direct participation on neuronal function (Garland et al., 2022). Therefore, to further explore the ET124 effects, we developed a novel advanced microfluidic system featuring human neuron–microglia–astrocyte tricultures, where SH-*SWE* cells and H_2_O_2_ (at low concentrations) were intended to recapitulate age-associated AD, as aforementioned. Several optimizations were necessary to establish functional tricultures in the tricompartmentalized microfluidic device. In addition to the definition of the most appropriate coating for the three cell types, we readjusted the media composition, as detailed in Material and methods. L-glutamine (a normal constituent in microglia media) was avoided due to potential side effects in the neuronal cells, including excitotoxicity and calcium dysregulation (Al-Nasser et al., 2022). Another optimization was the relative proportion of neurons/microglia/astrocytes in the tricultures. We employed 50% neurons, 30% astrocytes, and 20% microglia (5:3:2 ratio) that showed better stability during the 48 h of the triculture. The microfluidic device with microchannels of 5 µm in width, 2.5 µm in height, and 50/250 µm in length was fabricated (De Vitis et al., 2021) to allow the passage of neurites (more easily with 50 µm length for some specific studies), cell-derived growth factors, and exosomes, as ET124. Such reduced dimensions provided the optimal tool in only requiring a small number of cells and exosomes, in contrast with other proposed systems (Bruce et al., 2015; Hajal et al., 2022).

Inflammation, ageing, and oxidative stress are mutually perpetuated in AD, contributing to the disease progression. These features were reproduced in our age-AD model with the SH-*SWE* cells and mild H_2_O_2_ concentration (van Rensburg et al., 1997; Milton, 2004; Wang et al., 2023). The addition of H_2_O_2_ was shown to induce cell stress and senescence (Chadwick et al., 2010). Downregulation of miR-124-3p in the monocultured SH-*SWE* cells (Supplementary Figure S3) was not observed in the triculture system, suggesting the presence of compensatory mechanisms by the glial cells, which are known to protect neurons against oxidative stress (Iwata-Ichikawa et al., 1999).

Following the administration of ET124 into the triculture system, its higher accumulation in the microglial compartment confirms the suggested tropism of neuron-derived exosomes for microglia (Bahrini et al., 2015; Peng et al., 2021). Furthermore, our study revealed that ET124 prevents neuronal death and inhibits late apoptosis in the AD triculture system, maintaining viability levels comparable to control. Such neuroprotective effects of ET124 were further evidenced by mitigating caspase-12, a protein known to respond to both amyloid toxicity and H_2_O_2_ oxidative stress in the AD context (Nakagawa et al., 2000; Quiroz-Baez et al., 2011). Hence, such mitigation of caspase-12 in neurons from the AD triculture model treated with ET124 is elucidative of the neuroprotective potential of miR-124 in mitigating ER stress and related apoptotic pathways previously mentioned.

The activation of NF-κB, described as implicated in Aβ deposition, neuroinflammation, and neurodegeneration (Sun et al., 2022), was inhibited by ET124 in astrocytes, despite the pronounced translocation exhibited by neurons and microglia in the AD triculture system. Intriguingly, ET124 had limited impact on the expression of mitochondrial fusion and fission genes, as well as in the proliferation marker Ki-67. These findings underscore the mitochondria neuroprotective effects under a slight rise of mitochondrial ROS associated with preconditioned tolerance (Correia et al., 2010).

Upon acute or chronic stimulation, microglia undergo context- and temporal-dependent changes that modify their morphology and inflammatory signature, becoming activated (Clarke and Patani, 2020). Sometimes, such an activation profile can be neurotoxic by excessive neuroinflammation that impairs neurons and astrocytes (Brites, 2020). Indeed, microglia in our AD microfluidic tricultures exhibited such morphological changes, manifested by a decreased cell area and perimeter but an increased cell circularity. In addition, *TREM2* downregulation, together with the low P2RY12 immunofluorescence in microglia at the AD microfluidic chips, indicate impaired phagocytic capacity, activation, and metabolism (Akhter et al., 2021). In AD, TREM2 promotes the clearance of neuronal toxic products (Li et al., 2022), being essential for the response to Aβ plaque-induced pathology (Ulland and Colonna, 2018). Furthermore, microglia from the AD microfluidic triculture exhibited upregulation of *HLA-DRA* (MHCII coding gene), *ARG1*, and *S100B*, consistent with activation and adaptive immune cell characteristics. ET124 treatment effectively increased microglial miR-124 levels and prevented alterations in their morphology, as well as in the expression of *P2RY12*, *TREM2*, *HLA-DRA*, and *HMGB1*. Such effects minimized the AD-related molecular signature and supported the preservation of neuroreparative and functional microglia. Future studies should additionally investigate the benefits of ET124 on the accumulation of microglia and neuronal lipid droplets, as well as in the dysfunctional inflammasome–autophagy interplay, as characteristic AD features (Claes et al., 2021; Lu et al., 2022; Li et al., 2024).

Neuronal expression of miR-124 is known to mediate neurite outgrowth by regulating genes involved in cytoskeleton organization (Yu et al., 2008). Indeed, such effects were observed in the AD triculture system, with the ET124 treatment sustaining neurite outgrowth in terms of number, length, and transmigration into other compartments. Furthermore, increased neuronal *NOS1* expression in the AD triculture system recapitulated data from postmortem AD patient samples, characterized by increased oxidative stress and activated glia (Luth et al., 2002). Remarkably, ET124 not only counteracted such *NOS1* overexpression but also further extended their benefits by partially recovering the expression of presynaptic (*SYN*) and post-synaptic (*DLG4)* genes, potentially improving synaptic function that is critically impaired in AD (Heffernan et al., 1998; Sultana et al., 2010). ET124 also switched miR-146a expression from negative towards positive levels, in accordance with the direct correction found in previous studies from our group (Vaz et al., 2021).

Astrocytes, in combination with microglia, contribute to homoeostasis, immune response, BBB regulation, and synaptic dynamics. Communication between microglia and astrocytes influences and coordinates each other and their effects on neural function and disease (Garland et al., 2022). In AD, astrocytes undergo morphological, molecular, and functional changes, designated as astrogliosis, becoming harmful to both neurons and microglia (Smit et al., 2021). Our data in the AD microfluidic system robustly support an increased astrocyte reactivity, based on the altered morphological changes and augmented *GFAP*, *GJA1*, *AGER*, and *S100B* gene expression levels together with their correspondent protein immunofluorescence detection, commonly identified in AD (Monterey et al., 2021). Importantly, AD astrocytes revealed a marked downregulation of miR-124-3p, miR-146a-5p, and miR-125-5p, implicated in AD-associated reactive astrocytosis and observed in AD patients (Iyer et al., 2012; Yashoa and Nabi, 2022; Papadimitriou et al., 2023). It should be noted that astrocytic miR-124-3p/miR-125b-5p/miR-146a-5p showed to be intercorrelated. Specifically, these astrocytic miRNAs not only correlated with neuronal miR-146a-5p and miR-125b-5p but also with microglial miR-124-3p, supporting intercellular crosstalk. Remarkably, treatment with ET124 prevented morphological changes in AD astrocytes, counteracted S100B increased expression levels, and upregulated miR-124-3p/miR-146-5p/miR-125b-5p. The regulatory effects of ET124 were also observed in the downregulation of astrocytic miR-155-5p, whose increased expression in astrocytes is associated with a pro-inflammatory medium (Korotkov et al., 2020) and of miR-21-5p that was shown to be associated with multiple AD-associated pathologies (Kim et al., 2023). In summary, these data suggest ET124 immunoprotective effects on AD astrocytes.

**FIGURE 8:**
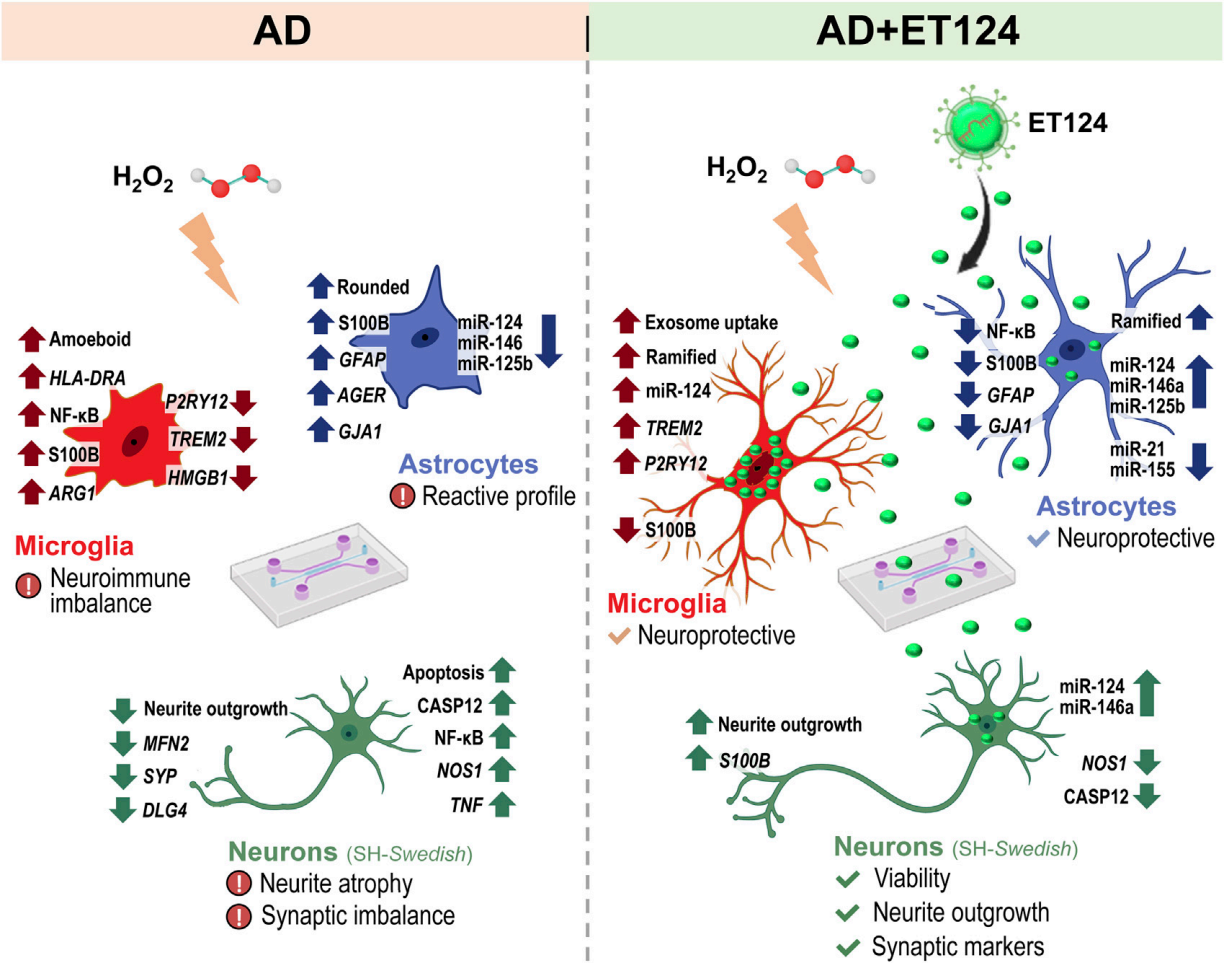
Schematic representation of the main alterations observed in the age-AD like model with microfluidic neural tricultures and the emerging role of miRNA-124-loaded exosomes (ET124) as novel therapeutics in Alzheimer’s disease (AD). Data were obtained from neuron–glia human tricultures in tricompartmentalized microfluidic devices, allowing communication between neuron-like (SH-WT human neuroblastoma cells in control, or SH-SWE in AD system), astrocytes, and microglia human cell lines (IM-HT and HMC3, respectively). The three cell types were simultaneously stressed with H_2_O_2_ (in AD system), co-treated with H_2_O_2_ + ET124 (in AD + ET124 system), while control devices did not receive any treatment. The age-AD model (left panel, AD) depicts the most relevant alterations obtained in neuron-like cells, as well as in astrocytes and microglia, in the AD triculture model, which are predominantly associated with glial activation, oxidative stress, and neuroinflammation (including the presence of inflammatory-associated miRNAs). Treatment with ET124 (right panel, AD + ET124) that triggered the upregulation of miRNA-124 in all cell types; prevented alterations in neurite outgrowth, synaptic imbalance, and cell demise; as well as the appearance of inflammatory markers and miRNAs, thus sustaining neuroprotective glial phenotypes. Therefore, ET124 reveals to be a potential therapeutic tool capable of fostering neuroprotective effects through its unique capacity to modulate the neuron–glia immune response in the context of AD. AD, Alzheimer’s disease; ET124, exosomes isolated from the SH-*WT* cell secretome after transfection with miR-124-3p mimic; SH-*WT, wild-type* human SH-SY5Y cells; neurons, SH-*SWE*, SH-SY5Y cells expressing the APP *Swedish* variant; Microglia (human CHME3 cell line); astrocytes; IM-HA, immortalized human astrocyte; NF-κB, nuclear factor kappa of activated B cells; S100B, S100 calcium-binding protein Β; *HLA-DRA*, major histocompatibility complex class II (MHC-II)-coding gene; *ARG1*, arginase-1-coding gene; *P2RY12*, purinergic P2Y12 receptor-coding gene; *TREM2*, triggering receptor expressed on myeloid cells 2-coding gene; *HMGB1*, high mobility group box protein 1-coding gene; *GFAP*, glial fibrillary acidic protein-coding gene; *AGER*, advanced glycation end product receptor-coding gene; *GJA1*, gap junction protein alpha 1 gene (coding for connexin 43); *MFN2*, mitofusin2 mitochondrial gene; *SYP*, synaptophysin-coding gene; *DLG4*, postsynaptic density protein 95 (PSD95)-coding gene; CASP12, caspase-12; *NOS1*, neuronal nitric oxide synthase-coding gene; *TNF*, tumour necrosis factor alpha-coding gene (Created with BioRender.com).

Collectively, this study shows that the direct transfection of neuronal exosomes with miR-124 (ET124) is more efficient than the cell transfection to obtain miR-124-loaded exosomes (CT124). Likewise, it also supports the therapeutic benefits of delivering ET124 into an age-AD *in vitro* triculture model by preserving neurite outgrowth and viability, as well as neuron–glial cell morphology and overexpression of inflammatory markers, including miRNAs, as schematized in Figure 8. It was also clear that ET124 are increasingly phagocytosed by the microglia from mouse cortical primary cultures than CT124, suggesting that direct transfection with miR-124 may cause exosomal membrane modifications that facilitate recognition by microglial cells, an issue to be explored in the upcoming future.

To approach the clinic translation, we are now using intra-orbital injection of ET124 in 9-month-old 5xFAD mice, which exhibited the most harmful interrelationships between miRNAs and gene expression levels associated with neurodegeneration and neuroinflammation (Ianni et al., 2024). Preliminary results suggest a recovery in spatial learning and memory in the animals injected with ET124 when compared to treatments with PBS alone or empty exosomes (unpublished data). Hippocampal and brain cortex samples already collected will further elucidate the regional aspect of the treatment and the eventual regeneration of the homoeostatic balance, following ET124 administration. Neuronal/glial/inflammatory markers selected from the Ianni et al., published study will be then investigated for preclinical validation of the miR-124-engineered neural exosomes. In addition to using the 5x FAD mice, we do not discard that additional models recapitulating AD pathogenesis may be required to enhance the robustness and reliability of this study conclusions. Moreover, numerous challenges should be addressed before bringing therapeutic miRNAs into clinical practice due to potential immunogenic reactions and off-target effects. Indeed, none of the miRNA-based therapeutics reached phase III clinical trials or was approved by the US Food and Drug Administration (FDA) so far (Seyhan, 2024). Finally, our innovative microfluidics-based triculture platform holds promise to assist as a detection tool in patient-centric stratification care, as recently proposed (Hauser et al., 2023), namely, in AD patients presenting innate immune activation or RNA dysregulation, recently categorized as pathophysiological subtypes 2 and 3, respectively (Tijms et al., 2020). Patients with defective miR-124 expression in neural cells (generated by transdifferentiation or iPSCs-derived), in monocultures, or in tricultures can then be treated with ET124. Ideally, in the future, ET124 can be even prepared from the patient cells to avoid treatment with immunosuppressors, although their applicability in clinical settings still requires further research. Microfluidic tricultures may additionally serve in the development of more precise immunotherapeutic agents, including exosome-based therapies, aligning with advancements in the precision medicine field and learning-assisted algorithmic analysis (Hua et al., 2024).

## 5 Conclusion

As far as we know, this research pioneers the impact of miR-124-engineered exosomes (ET124) in regulating the interactions between neurons, microglia, and astrocytes in an age-AD model supported by a microfluidic triculture system. The study offers novel proof, concerning the role of miR-124-loaded exosomes as an innovative tool in neurodegenerative diseases and highlights their therapeutic potential for AD by attenuating/suppressing neurodegeneration and neuroinflammation, as well as by potentially preventing disease dissemination through paracrine signalling regulation. In future studies, we will address the underlying mechanisms of ET124 treatment (and potentially other exosome-based formulations) in the 5xFAD mouse model and AD patient iPSC-derived brain cells. Lastly, the present study provides a prognostic assessment tool that can be used for patient-personalized medicine and as a foundation for future exosome-based therapeutical strategies and their clinical applications for counteracting AD progression.

## Data Availability

The original contributions presented in the study are included in the article/Supplementary Material; further inquiries can be directed to the corresponding author.
