# Supplementary material for: Exosomes enriched with miR-124-3p show therapeutic potential in a new microfluidic triculture model that recapitulates neuron–glia crosstalk in Alzheimer’s disease
Source: Front Pharmacol. 2025 Mar 12;16:1474012. doi: 10.3389/fphar.2025.1474012 (PMC11936931; doi:10.3389/fphar.2025.1474012)
Supplement: Supplementary file 1 [file DataSheet1.pdf]

Supplementary Material:

Exosomes enriched in miR-124-3p show therapeutic potential in a new microfluidic triculture model that recapitulates neuron-glia crosstalk in Alzheimer's disease

Artemizia Évora[1†], Gonçalo Garcia[1,2†], Ana Rubi[1], Eleonora De Vitis[3], Ana Teresa Matos[1], Ana Rita Vaz[1,2], Francesca Gervaso[3], Giuseppe Gigli[3,4], Alessandro Polini[3], Dora Brites[1,2*]

[1]Neuroinflammation, Signaling and Neuroregeneration, Research Institute for Medicines (iMed.ULisboa), Faculty of Pharmacy, Universidade de Lisboa, Lisbon, Portugal
[2]Department of Pharmaceutical Sciences and Medicines, Faculty of Pharmacy, Universidade de Lisboa, Lisbon, Portugal
[3]Institute of Nanotechnology, National Research Council (CNR Nanotec), Lecce, Italy
[4]Dipartimento di Medicina Sperimentale, Università Del Salento, Lecce, Italy

[†]These authors contributed equally to this work and share first authorship

***Correspondence:**
Dora Brites
dbrites@ff.ulisboa.pt

Supplementary Table 1. List of the primers used in RT-qPCR to amplify miRNAs.

miRNA	species	Target sequence (5’-3’)
miR-124-3p	*hsa/mmu*	UAAGGCACGCGGUGAAUGCC
miR-125b-5p	*hsa/mmu*	UCCCUGAGACCCUAACUUGUGA
miR-21-5p	*hsa/mmu*	UAGCUUAUCAGACUGAUGUUGA
miR-146a-5p	*hsa/mmu*	UGAGAACUGAAUUCCAUGGGUU
miR-155-5p	*hsa*	UUAAUGCUAAUCGUGAUAGGGGU
SNORD110	*hsa/mmu*	Reference gene
U6	*hsa/mmu*	Reference gene
UniSp6 (spike-in)	*hsa/mmu*	Reference gene

hsa, *homo sapiens*; *mmu*, *mus musculus*; miR, microRNA; RT-qPCR, reverse transcription-quantitative polymerase chain reaction.

Supplementary Table 2. List of primer sequences used in RT-qPCR to amplify protein-coding genes.

Gene	species	Forward primer sequence (5'-3')	Reverse primer sequence (5'-3')
ACTB	*hsa*	CAGAGCCTCGCCTTTGCCGA	ATCCATGGTGAGCTGGCGGC
Actb	*mmu*	GCAGGAGTACGATGAGTCCG	ACGCAGCTCAGTAACAGTCC
AGER	*hsa*	GCCACTGGTGCTGAAGTGTA	GTCCGGCCTGTGTTCAGTTT
APP_{wt}	*hsa*	AGGAGATCTCTGAAGTGAAGA	GCACCTTTGTTTGAACCCAC
APP_{swe}	*hsa*	AGGAGATCTCTGAAGTGAATC	GCACCTTTGTTTGAACCCAC
ARG1	*hsa*	ACTGAGGGTTGACTGACTGGA	TTCTTCCACCCCTCCTCGTG
Arg1	*mmu*	CTTGGCTTGCTTCGGAACTC	GGAGAAGGCGTTTGCTTAGTTC
DLG4	*hsa*	CATTGGAAAGGGGTAACTCAG	AATGATCTTGGTGATGAAAATG
FIS1	*hsa*	CCAAGAGCACGCAGTTTGAG	ACGTAATCCCGCTGTTCCTC
GFAP	*hsa*	GAGGTTGAGAGGGACAATCT	GCTTCATCTGCTTCCTGTCT
GJA1	*hsa*	GTTCAATCACTTGGCGTGAC	AGTTGAGTAGGCTTGAAC
HLA-DRA	*hsa*	AGGGATTGCGCAAAAGCA	TCACCTCCATGTGCCTTACAGA
HMGB1	*hsa*	TTGTGCAAACTTGTCGGGAG	TTAGCAGACATGGTCTTCCACC
MFN2	*hsa*	AGCCAACCTCAACCTGAGAC	GCATCGAGAGAAGAGCAGGG
MKI67	*hsa*	TCCTTTGGTGGGCACCTAAGACCTG	TGATGGTTGAGGCTGTTCCTTGATG
NOS1	*hsa*	CCTGGCCAATGTGAGGTTCT	AGCTCATCCCCTTCCCTCAT
NOS2	*hsa*	CAGCGGGATGACTTTCCAA	AGGCAAGATTTGGACCTGCA
Nos2	*mmu*	ACCCACATCTGGCAGAATGAG	AGCCATGACCTTTCGCATTAG
P2RY12	*hsa*	CCACTCTGCAGGTTGCAATA	TGCATTTCTTGTTGGTTACCTGA
S100B	*hsa*	TGTAGACCCTAACCCGGAGG	TGCATGGATGAGGAACGCAT
SYP	*hsa*	AGTGCGCTAGAGCATTCTGG	CCACCATTCTGCCTCGCTTA
TNF	*hsa*	AACCTCCTCTCTGCCATC	ATGTTCGTCCTCCTCACA
TREM2	*hsa*	ATGATGCGGGTCTCTACCAGTG	GCATCCTCGAAGCTCTCAGACT
Trem2	*mmu*	AACTTCAGATCCTCACTGGACC	CCTGGCTGGACTTAAGCTGT

hsa, Homo sapiens; mmu, Mus musculus; *RT-qPCR*, real-time quantitative polymerase chain reaction; *ACTB*/*Actb*, *β-actin* coding gene; *AGER*, advanced glycosylation end-product specific receptor coding gene; APP, Amyloid precursor protein coding gene (with *wild-type* and *Swedish* variants); *ARG1*/Arg1, arginase-1 coding gene; *DLG4*, postsynaptic density protein 95 (PSD95) coding gene; *FIS1*, fission 1 mitochondrial gene; *GFAP*, glial fibrillary acidic protein coding gene; GJA1 (*CX-43*), gap junction protein alpha 1 gene (connexin 43); *HLA-DRA*, major histocompatibility complex class II (MHC-II) coding gene; *HMGB1*, high mobility group box protein 1 coding gene; *MFN2,* mitofusin2 mitochondrial gene; M*KI67*, Ki-67 coding gene; *NOS1*, neuronal nitric oxide synthase coding gene; *NOS2/Nos2*, inducible nitric oxide synthase coding gene; *P2RY12*, purinergic P2Y12 receptor coding gene; *S100B*, S100 calcium-binding protein B coding gene; *SYP*, synaptophysin coding gene; T*NF*, tumour necrosis factor alpha coding gene; *TREM2/Trem2*, triggering receptor expressed on myeloid cells 2 coding gene.

Supplementary Table 3. List of antibodies used in the immunocytochemistry assays.

Primary antibodies	Source, Ref#	Species	Dilution
Caspase-12	Sigma, C7611	Rat	1:100
IBA1	Invitrogen, MAS-27726	Mouse	1:100
MAP-2	Millipore, MAP3418	Mouse	1:100
NF-κB p65	BioLegend, 622602	Rabbit	1:100
P2RY12	BioLegend, 848002	Mouse	1:100
S100β	AbCam, ab52642	Rabbit	1:100
βIII-Tubulin	Millipore, MAB1637	Rat	1:100
**Secondary antibodies**			
**Alexa 405 anti-rabbit**	Invitrogen, A31556	Goat	1:250
**Alexa 488 anti-mouse**	Invitrogen, A32723	Goat	1:500
**Alexa 647 anti-mouse**	Invitrogen, A21236	Goat	1:500
**Alexa 594 anti-rat**	Invitrogen, A21209	Donkey	1.500

IBA1, ionized calcium-binding adapter molecule; *MAP-2,* microtubule associated protein 2; *NF-κB p65,* Nuclear Factor kappa of activated B cells 65kDa subunit; *P2RY12,* purinergic P2Y12 receptor; *S100B,* S100 calcium-binding protein B.

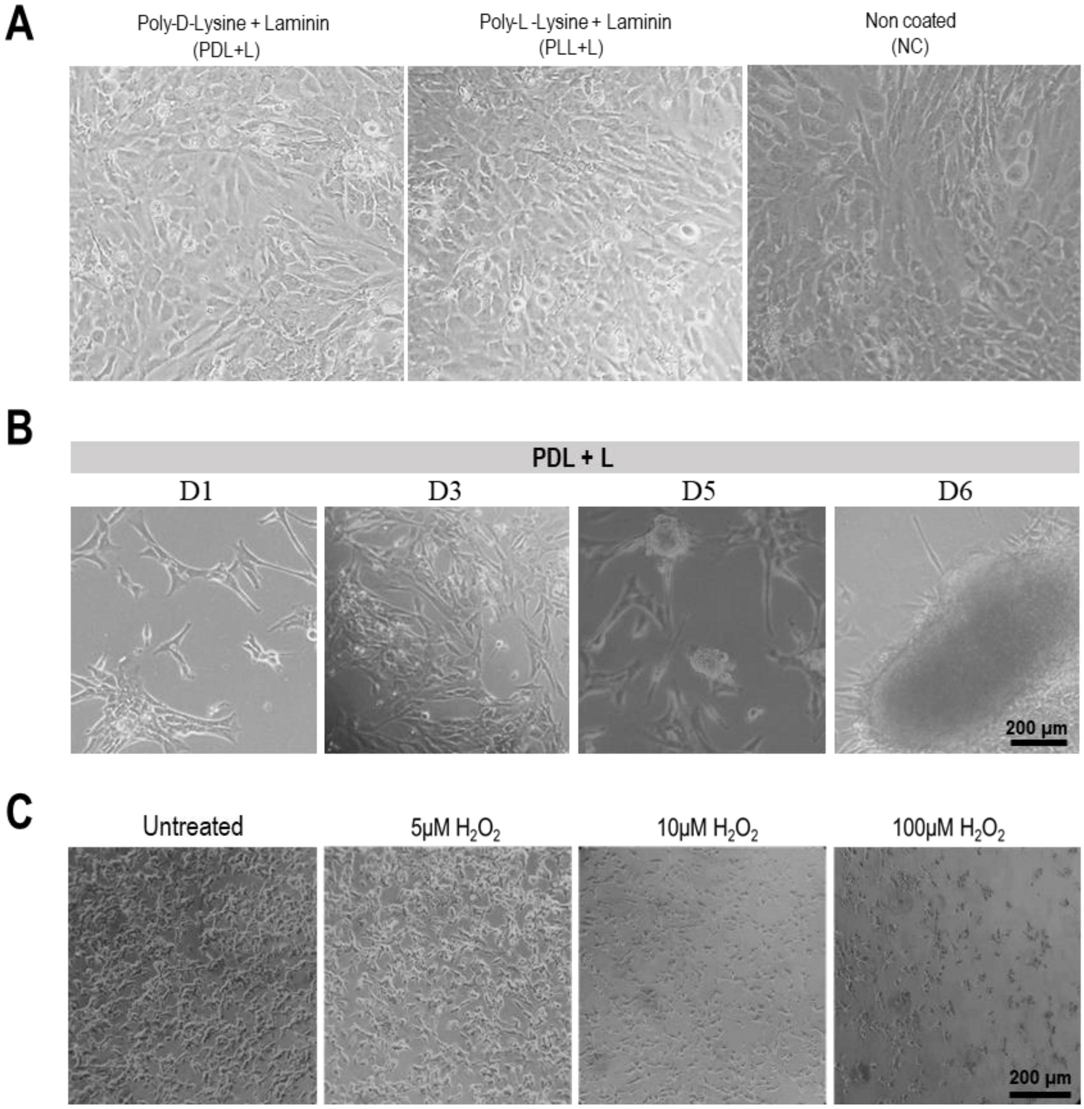

Supplementary Figure 1. Comparison of astrocyte adherence and growth using the different coating formulations. (**A**) Phase-contrast images showing that immortalized human astrocytes (IM-HA) equally grow in different coating setups: Poly-D-Lysine & Laminin (PDL+L), Poly-L-Lysine & Laminin (PLL+L) or non-coated (NC). To note, however, that astrocytes tend to form spheroids if coated for more than 5 days in PDL+L **(B)**. Thus, PDL+L was decided to be employed in the triculture system, where astrocytes were cultured for 3 days. (**C**) Testing different H_2O_2 concentrations (5, 10 and 100 µM) in SH-SY5Y neuroblastoma cells showed no apparent cell death up to 10 µM. D, days *in vitro*.

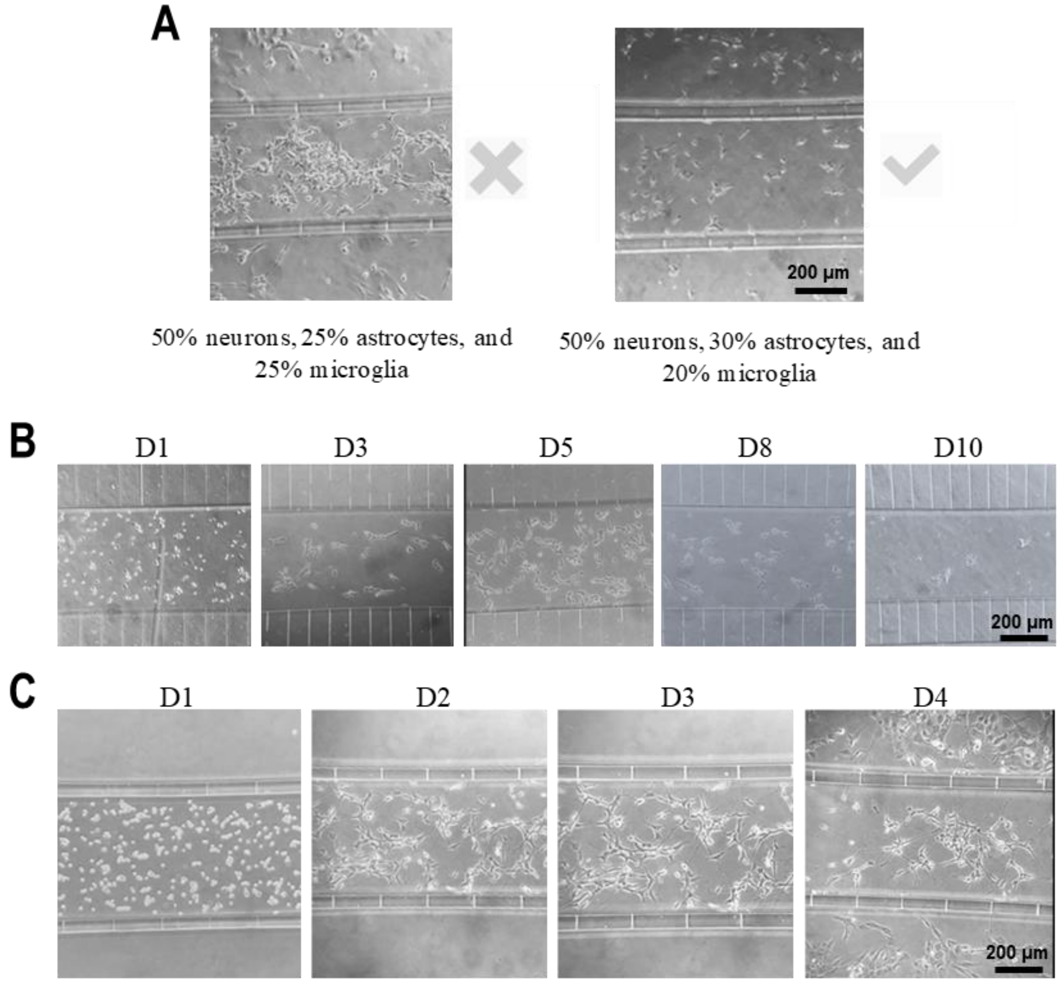

Supplementary Figure 2. Neurons-astrocytes-microglia at a cell ratio of 5:3:2, respectively, showed to be the most suitable condition for a sustained Triculture Microfluidic System for 5 days. (**A**) Two different cell ratios were tested (50% neurons, 30% astrocytes, and 20% microglia; 50% neurons, 25% astrocytes, and 25% microglia) using Poly-D-lysine + Laminin (PDL+L) coated in the tricompartmental microfluidic device. The right panel evidenced the best cell ratio for a healthful neural cell triculture. Here, we used the devices with microchannels of 5 µm diameter and 50 µm long to favour extended intercellular communication and better decide on the cell type ratio for the subsequent experiments. (**B**) Until the 5 days of retinoic acid (RA) differentiation, the adherence of SH-WT cells in the system were optimal. Extension of neurites to other compartments was hampered by the microchannels with 250 µm long. At 8 and 10 days of culture, detachment of RA-differentiated neurons was noticed. (**C**) Time-lapse images of neuronal RA-differentiation for the selected 3 days in culture before the platting of human microglia (CHME3) and astrocytes (immortalized human astrocytes) in the microfluidic triculture system. The interaction with neighbouring glial cells facilitated by the 5 µm diameter and 50 µm long microchannels appeared to favour neuronal differentiation in the system. SH-*WT, wild-type* human SH-SY5Y cells were used as neuron-like cells. D, days *in vitro*.

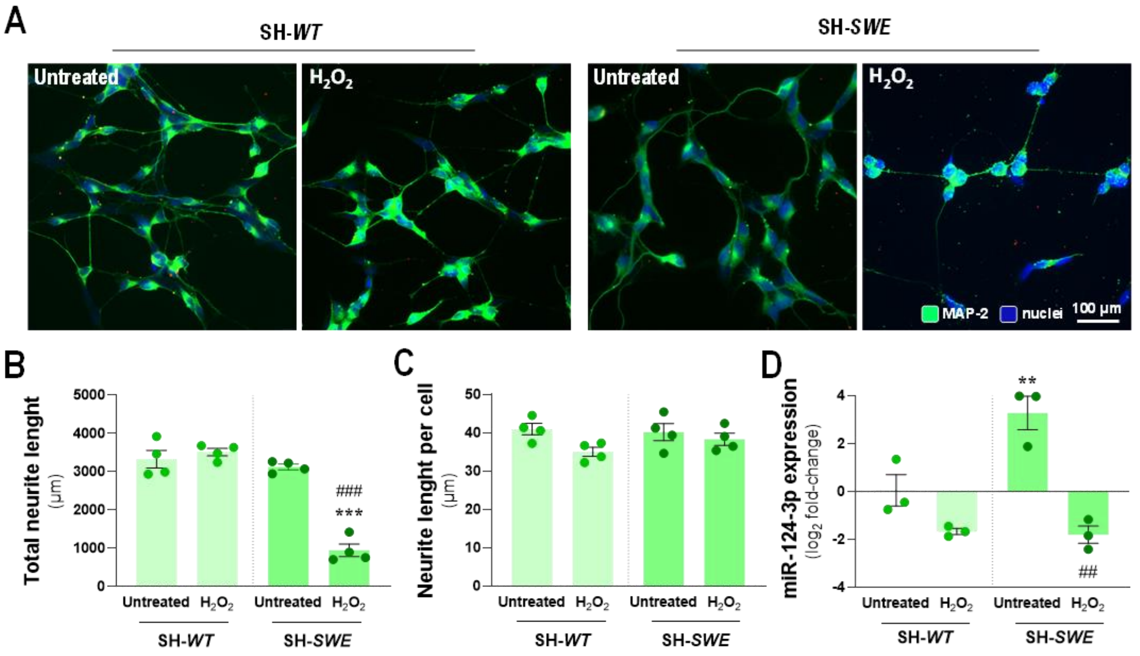

Supplementary Figure 3. Differential susceptibility of SH-*WT* and SH-*SWE* cells to H_2O_2-induced oxidative stress, ageing and neuroinflammation. (**A**) Representative fluorescence images of MAP2 in differentiated SH-*WT* and SH-*SWE* cells. Effects of H_2O_2 (10 µM) in total neurite outgrowth (**B**), neurite length per cell (**C**) and miR-124-3p levels (**D**) in both SH-*WT* and SH-*SWE* cells. SH-*WT* and SH-*SWE* neuroblastoma cells were differentiated with retinoic acid, before being treated with 10 µM H_2O_2 for 24 h. Neurite outgrowth and length were measured using Aivia AI Image Analysis Software (Leica Microsystems). Expression of miR-124-3p was assessed by reverse transcription quantitative polymerase chain reaction, RT-qPCR. Data are mean ±SEM, from at least three independent experiments. ***$p<0.001$ and **$p<0.01$ *vs.* SH-*WT* cells; $^{###}p<0.001$ and $^{##}p<0.01$ *vs.* untreated cells, respectively, determined by one-way ANOVA with Tuckey post-hoc test. MAP2, microtubule associated protein 2. SH-*WT, wild-type* human SH-SY5Y cells; SH-*SWE*, SH-SY5Y cells expressing the APP *Swedish* variant.

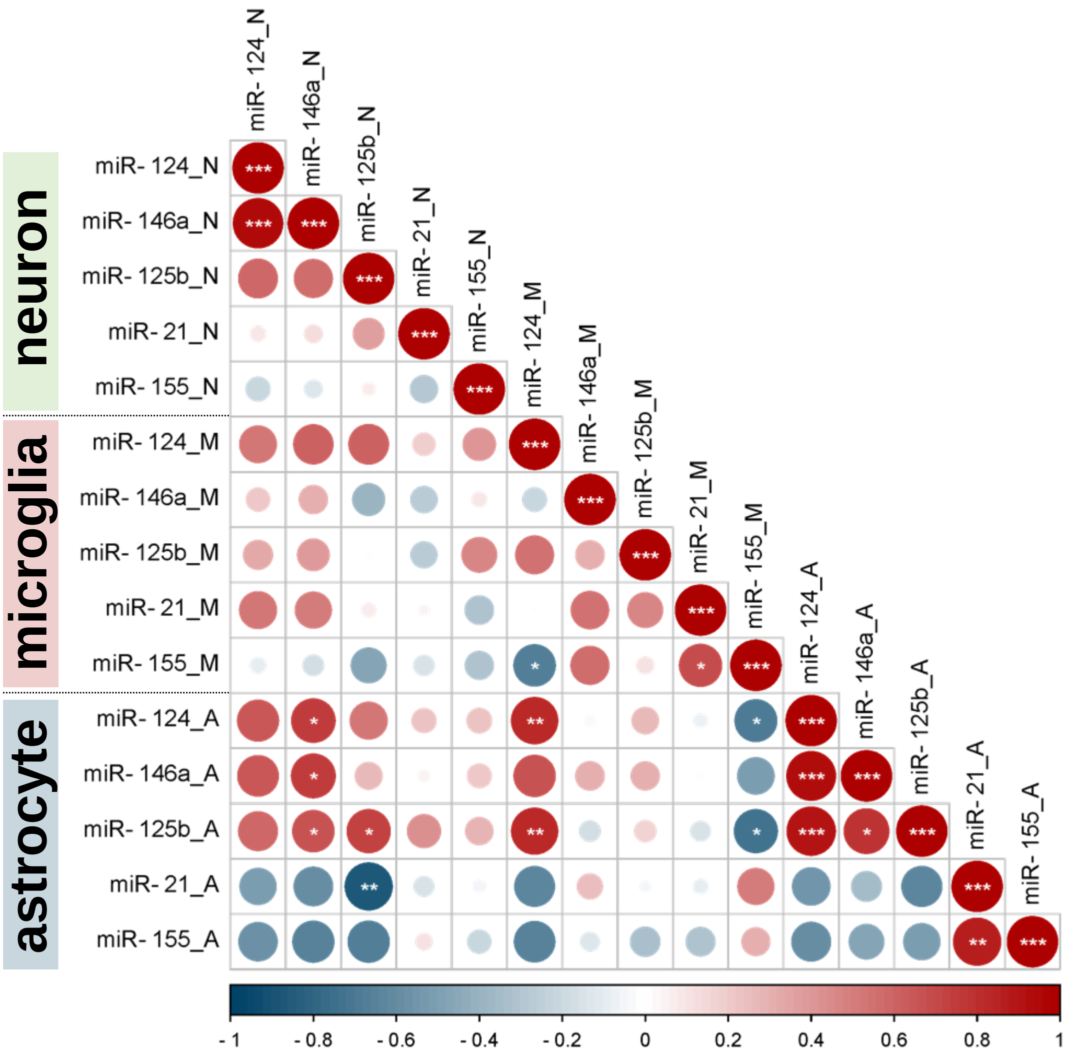

Supplementary Figure 4. Pearson's correlation matrix illustrates the pairwise relationships of commonly expressed inflammatory-associated miRNAs in the different microfluidic triculture systems (Control, AD, and AD+ET124). Each system comprised human neuroblastoma cells (SH-*WT* in Control, and SH-SWE in AD), microglia (human CHME3 cell line), and astrocytes (IM-HA, immortalized human astrocytes). In AD systems cells were stressed with H_2O_2; while in AD+ET124 systems cells were co-treated with H_2O_2 + ET124. Control systems did not receive any treatment. Colour mapping represents the correlation coefficient (R^2). Negative correlations are shown in blue and positive correlations in red, both integrating a respective annotation whenever significant. ***$p<0.001$, **$p<0.01$, *$p<0.05$. N, neuron-related; M, microglia-related; A, astrocyte-related; miR, microRNA; SH-*WT*, *wild-type* human SH-SY5Y cells; SH-*SWE*, SH-SY5Y cells expressing the *APP* Swedish variant.
